# Genetic Conservation of CBS Domain Containing Protein Family in *Oryza* Species and Their Association with Abiotic Stress Responses

**DOI:** 10.3390/ijms23031687

**Published:** 2022-02-01

**Authors:** Surabhi Tomar, Ashish Subba, Meenu Bala, Anil Kumar Singh, Ashwani Pareek, Sneh Lata Singla-Pareek

**Affiliations:** 1 Plant Stress Biology Group, International Centre for Genetic Engineering and Biotechnology, New Delhi 110067, India; tomar.surabhi@gmail.com (S.T.); ashishsubba11@gmail.com (A.S.); 2 School of Genetic Engineering, ICAR-Indian Institute of Agricultural Biotechnology, Ranchi 834010, India; balameenu.9@gmail.com (M.B.); anils13@gmail.com (A.K.S.); 3 ICAR-National Institute for Plant Biotechnology, LBS Centre, Pusa Campus, New Delhi 110012, India; 4 Stress Physiology and Molecular Biology Laboratory, School of Life Sciences, Jawaharlal Nehru University, New Delhi 110067, India; ashwanip@mail.jnu.ac.in; 5 National Agri-Food Biotechnology Institute, Mohali 140306, India

**Keywords:** Cystathionine β-synthase, CDCPs, *Oryza* species, promoter analysis, stress-responsive genes, wild rice

## Abstract

Crop Wild Relatives (CWRs) form a comprehensive gene pool that can answer the queries related to plant domestication, speciation, and ecological adaptation. The genus ‘*Oryza’* comprises about 27 species, of which two are cultivated, while the remaining are wild. Here, we have attempted to understand the conservation and diversification of the genes encoding Cystathionine β-synthase (CBS) domain-containing proteins (CDCPs) in domesticated and CWRs of rice. Few members of CDCPs were previously identified to be stress-responsive and associated with multiple stress tolerance in rice. Through genome-wide analysis of eleven rice genomes, we identified a total of 36 genes encoding CDCPs in *O. longistaminata*, 38 in *O. glaberrima*, 39 each in *O. rufipogon*, *O. glumaepatula*, *O. brachyantha*, *O. punctata*, and *O. sativa* subsp. *japonica*, 40 each in *O. barthii* and *O. meridionalis*, 41 in *O. nivara*, and 42 in *O. sativa* subsp. *indica*. Gene duplication analysis as well as non-synonymous and synonymous substitutions in the duplicated gene pairs indicated that this family is shaped majorly by the negative or purifying selection pressure through the long-term evolution process. We identified the presence of two additional hetero-domains, namely TerCH and CoatomerE (specifically in *O. sativa* subsp. *indica*), which were not reported previously in plant CDCPs. The in silico expression analysis revealed some of the members to be responsive to various abiotic stresses. Furthermore, the qRT-PCR based analysis identified some members to be highly inducive specifically in salt-tolerant genotype in response to salinity. The *cis*-regulatory element analysis predicted the presence of numerous stress as well as a few phytohormone-responsive elements in their promoter region. The data presented in this study would be helpful in the characterization of these CDCPs from rice, particularly in relation to abiotic stress tolerance.

## 1. Introduction

With a constant rise in the annual global rice consumption (about 504.3 million metric tons in the year 2020–2021), there is a need to increase its production to feed the rising world population, which is estimated to reach more than 9 billion by 2050 [1,2]. However, the climate change, soil degradation, and sensitivity of rice crops to various biotic and abiotic stresses pose a major challenge to meet this demand. In this scenario, to understand the mechanism of abiotic stress tolerance in rice, our lab previously carried out a comparative study between the transcriptome of salt-tolerant Pokkali and salt-sensitive IR64 rice genotypes, and identified many genes which showed differential regulation under salt stress treatment. Among these are the genes encoding members of a protein family containing the conserved Cystathionine β-Synthase (CBS) domain [3]. Subsequently, our lab carried out the genome-wide identification of the CBS domain containing proteins (CDCPs) in rice (*Oryza sativa* subsp. *japonica*) and *Arabidopsis* [4], and identified two CDCP members to impart tolerance to multiple abiotic stresses when overexpressed constitutively in tobacco [5,6].

The CBS domain, first identified by Bateman [7], consists of about 60 amino acid residues, and is conserved across all kingdoms of life. This domain generally exists as tandem repeats, mostly in pairs or quads, in the polypeptide. While some CDCPs are composed of only CBS domains, others possess additional hetero-domain(s). CBS domains are known to possess an affinity for various ligands, mainly the adenosine nucleotides, and have regulatory functions based on ligand-induced conformational changes [8,9]. Mutations in the CBS domain of different CDCPs in humans have been identified to be associated with several hereditary disorders [10,11], which implies an indispensable role of CBS domains or broadly CDCPs. However, despite such clinical significance, the studies on CDCPs have remained scarce in the plant kingdom.

Few groups working on plant CDCPs have shown the involvement of CDCP members in diverse functions, e.g., Degenerated Panicle and Partial Sterility (DPS1)/OsCBSDUF1, a CDCP member from rice, has been recently reported to have a role in ROS-dependent cuticle development in leaf and anther, and in regulation of the leaf senescence [12,13]. The overexpression of soybean CDCP genes, namely *GmCBS21* and *GmCBSDUF3*, have been reported to impart tolerance to low nitrogen and multiple abiotic stresses, respectively [14,15]. In *Arabidopsis*, AtCBSX1 and AtCBSX3 have been identified to interact with the thioredoxins (Trxs) in the chloroplast and mitochondria, respectively, and thereby regulate the ROS levels in the respective organelles [16,17]. The chloride channel sub-family of CDCPs in plants has been inferred to function in nitrate transport and Cl^−^ sequestration [18].

**Figure 1:**
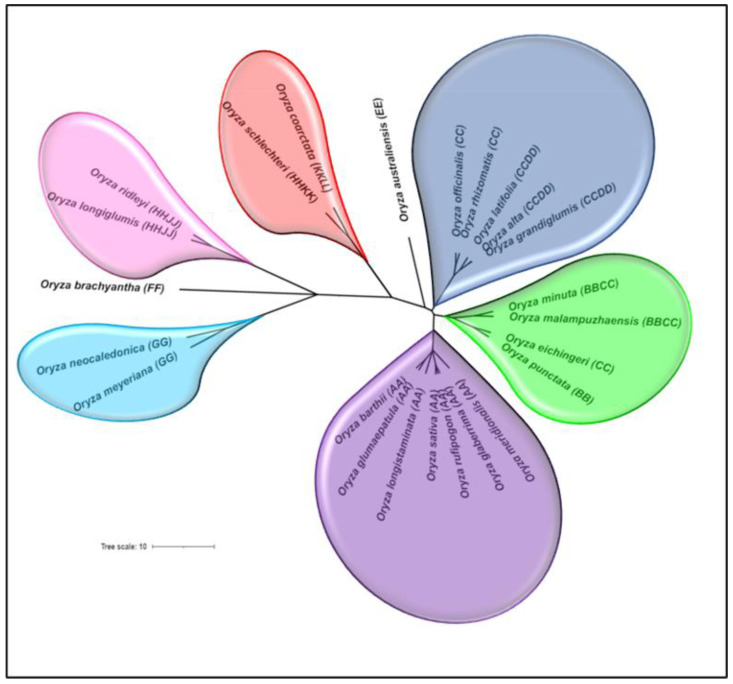
Phylogenetic tree depicting the evolution of different *Oryza* species. The representative species possessing each genome type, namely AA, BB, CC, EE, FF, GG, BBCC, CCDD, HHJJ, HHKK, and KKLL, including the ones with the completely sequenced genome (analyzed in this study) have been incorporated into the tree. The figure was generated using the TimeTree database (http://www.timetree.org/, accessed on 21 October 2021) and visualized using iTol (https://itol.embl.de/upload.cgi, accessed on 21 October 2021).

In the field of crop breeding, it has been realized that improvement of most of the major crops, including rice, has reached their plateau due to the narrow genetic base of the cultivated species. Hence, the researchers are gradually turning their attention towards the exploration and utilization of Crop Wild Relatives (CWRs), which encompasses a wide reservoir of genetic diversity valuable for the improvement of the crop, particularly for tolerance to different abiotic as well as biotic stresses with enhanced yield potential [19,20,21]. The genus ‘*Oryza*’ comprises about 27 species having distinct ecological adaptations. These include two independently domesticated cultivated rice species, viz., *O. sativa* (Asian rice) and *O. glaberrima* (African rice), whereas the remaining species are wild. These 27 *Oryza* species are estimated to have evolved over 15 million years ago and have diverged into 11 genome types comprising six diploid (AA, BB, CC, EE, FF, and GG) and five polyploid (BBCC, CCDD, HHJJ, HHKK, and KKLL) genomes [1,19]. The phylogenetic relationship among the *Oryza* species with different genome types is depicted in Figure 1. Both the cultivated rice species are diploid (AA genome), which have evolved through a series of events, such as introgression, natural selection, and breeding [22]. Therefore, considering the importance of CDCPs, particularly in conferring abiotic stress tolerance in plants, the present study was aimed to identify the CDCP families in the available genomes of ten *Oryza* species, comprising both wild and cultivated rice, to understand their evolution, conservation/diversification as well as their association with abiotic stress tolerance.

## 2. Results and Discussion

### 2.1. Number of Genes Encoding CDCPs Varies in Oryza Species

The whole-genome analysis for the genes encoding the CBS domain (Pfam id: PF00571) containing proteins (CDCPs) in 10 different *Oryza* species using the previously annotated sequences of CDCPs (encoded by 37 genes) from *O. sativa* subsp. *japonica* as queries [4] identified a total of 36 CDCP genes in *O. longistaminata*, 38 in *O. glaberrima*, 39 each in *O. rufipogon*, *O. glumaepatula*, *O. brachyantha*, and *O. punctata*, 40 each in *O. barthii* and *O. meridionalis*, 41 CDCPs in *O. nivara*, and 42 in *O. sativa* subsp. *indica*. Moreover, we also re-analyzed the genome sequence of *O. sativa* subsp. *japonica* and identified two new genes encoding CDCPs, unannotated in the earlier study by Kushwaha et al. [4], thus making a total of 39 CDCP genes in this species (Table 1). We classified these newly identified CDCPs from all 10 species into different subfamilies based on the presence of CBS domains in pairs or quads, the presence or absence of other associated hetero-domain(s) in the proteins, and their sequence identity with the ones from *O. sativa* subsp. *japonica*, which has been annotated by Kushwaha et al. [4]. Notably, we have incorporated some changes for updating in the previously classified order of CDCPs by Kushwaha et al. [4], specifically in the protein members containing only one pair or two pairs of CBS domains (CBSX and CBSCBS, respectively, discussed in the following paragraphs).

**Figure 2:**
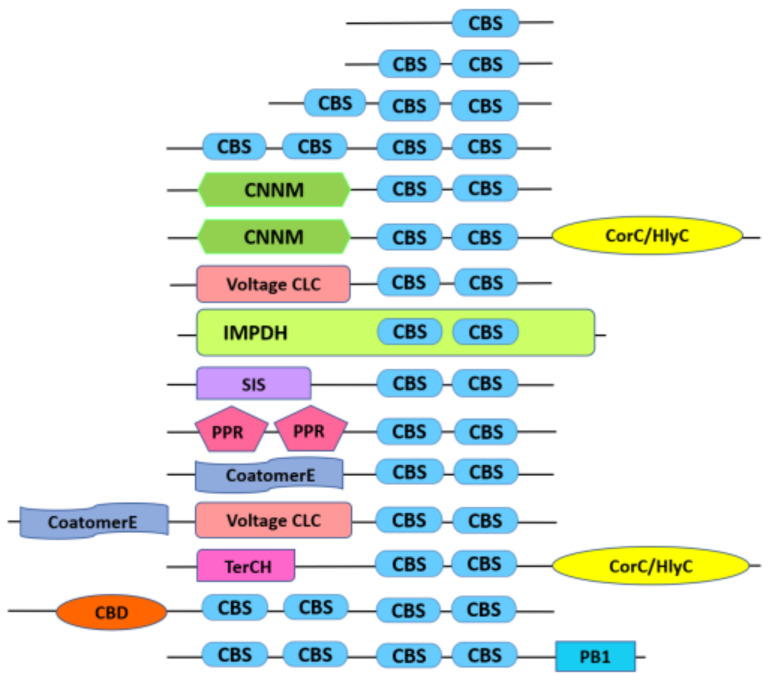
Schematic representation (unscaled) of the domain organization of different CDCPs in rice. CBS domains have been found to occur alone as well as in association with other domains in the polypeptide. ‘CBS’ denotes a CBS domain. Other domains include CNNM (or DUF21), CorC_HlyC, voltage chloride channel (Voltage CLC), IMPDH, Sugar isomerase (SIS), Pentatricopeptide repeat (PPR), Phox and Bem1(PB1), Carbohydrate binding domain (CBD) Coatomer epsilon subunit (CoatomerE), and TerCH. Note that in IMPDH, a pair of CBS domains occurs within the IMPDH domain.

In addition to different hetero-domains reported earlier by Kushwaha et al. [4] in the CDCPs from *O. sativa* subsp. *japonica*, which includes the CNNM/DUF21 (PF01595), CorC_HlyC (PF03471), Chloride Channel (CLC; PF00654), Inosine-5′-Monophosphate Dehydrogenase (IMPDH; PF00478), Sugar Isomerase (SIS; PF01380), Pentatricopeptide Repeat (PPR; PF01535), and Phox and Bem1 (PB1; PF00564) domains, three new domains, namely Coatomer epsilon subunit (CoatomerE; PF04733), terC (PF03741), and Carbohydrate binding domain (CBD; PF16561), were also found to be present in some CDCP members from *Oryza* species (Figure 2). The CoatomerE domain existed in association with a pair of CBS domains in *O. sativa* subsp. *indica* (OsICBSCoatomer E), while in *O. longistaminata*, this domain was found to be present in chloride channel members of CDCPs (OlCBSCLC5). In yeast and mammals, the CoatomerE domain is found among the seven subunits of Coat Protein Complex1 (COP1) [23], which is involved in the early retrograde transport of proteins from Golgi to Endoplasmic Reticulum as well as in intra Golgi transport [24]. The TerCH domain, involved in natural resistance to xenobiotic compounds in bacteria [25], was found only in *O. sativa* subsp. *indica*, in association with the CBS_CorC_HylC domain at the C-terminus. In *Arabidopsis*, a protein containing the lone TerCH domain has been reported to participate in the thylakoid membrane biogenesis and the *de novo* synthesis of the Photosystem II core proteins, while its knockout resulted in chlorophyll-deficient lethal seedlings [26,27].

Previously, Kushwaha et al. [4] used Pfam (version 21.0) for their analysis as well as the systematic classification of the CDCPs. However, with the rapid advancements and up-gradation of different tools, the classification needed to be modified and updated as per the latest predictions. We, therefore, made some changes in the pre-existing classification and the same has been used for all the subsequent analyses. The re-classification for different CDCPs from *Oryza* species has been systematically arranged and presented in Table 1. The previously reported CBSX7 and CBSCBS1 are the products of a single gene (LOC_Os01g40420) in *O. sativa* subsp. *japonica*, of which the longest isoform (CBSCBS1) harbors two pairs of CBS domains. As a result, we have reclassified it and its homologs in other *Oryza* species as CBSCBS1. Additionally, the previously reported CBSCBS5 has now been predicted to possess a single pair of CBS domains, and therefore, we have reclassified it as the new CBSX7. Additionally, we observed the carbohydrate binding domain in two members of CDCPs, which were earlier classified as CBSX8 and CBSCBS4 by Kushwaha et al. [4]. These two proteins have now been renamed as CBSCBSCBD1 and CBSCBSCBD2, respectively, to distinguish them from CBSCBS members. CBSCBSCBD2 was observed to be absent in *O. brachyantha*. The orthologs of CBSCBSCBD in *Arabidopsis* have been reported to function as hybrid βγ-subunits of SnRK complexes [28] which regulate various cellular processes, including plant growth and stress responses [29]. Consequently, the newly identified CDCP genes in *O. sativa* subsp. *japonica* with the locus id LOC_Os08g41740 and its respective homologs in other *Oryza* species have now been classified as *CBSX8.* The previously named CBSX10 protein was found to possess two pairs of CBS domains; hence, it has been renamed as the new CBSCBS4. Another newly identified CDCP (gene id: LOC_Os10g35630) and its homologs in other *Oryza* species possess a pair of CBS domains and have now been classified as CBSX10.

We noticed the presence of CBS domains either in pairs or quads in all the CDCPs from different *Oryza* species, except in the genome of *O. meridionalis* and *O. nivara*, where we found two genes (gene id: OMERI02G33320 and ONIVA05G14030, respectively) that encodes for a protein containing only one CBS domain, and a gene in *O. meridionalis* (OMERI05G12070) to encode a protein containing three CBS domains. We anticipated the possible loss of one CBS domain from these proteins during the course of evolution and named the former two as OmCBSX13 and OnCBSX15, respectively, while the latter containing three CBS domains is classified as OmCBSCBS5.

The differences in the total number of genes encoding CDCPs across 11 genomes from 10 *Oryza* species were observed to be mainly due to the gain or loss of *CBSX* and/or *CBSCBS* members during evolution (Table 1). However, *CBSCLCs* showed high conservation in all these species, except in *O. meridionalis*, where two *CBSCLCs* were found to be absent, while *O. barthii* possessed an additional *CBSCLC*. The absence of a member of either *CBSDUF* or *CBSCBSPB* sub-family was also observed in five *Oryza* species, namely *O. sativa* subsp. *japonica*, *O. barthii*, *O. glaberrima*, *O. rufipogon*, and *O. meridionalis*. Interestingly, *CBSIMPDH*, *CBSSIS*, *CBSPPR*, and *CBSDUFCH* were found to exist as a lone gene, but conserved in the genome of all the *Oryza* species studied, except *O. glaberrima*, which contains two members of *CBSDUFCH*, and *O. longistaminata*, in which *CBSSIS1* was annotated to encode a truncated protein without CBS domain, so it was not included in the present study.

### 2.2. Phylogenetic Analysis of CDCPs in Cultivated and Wild Rice

**Figure 3:**
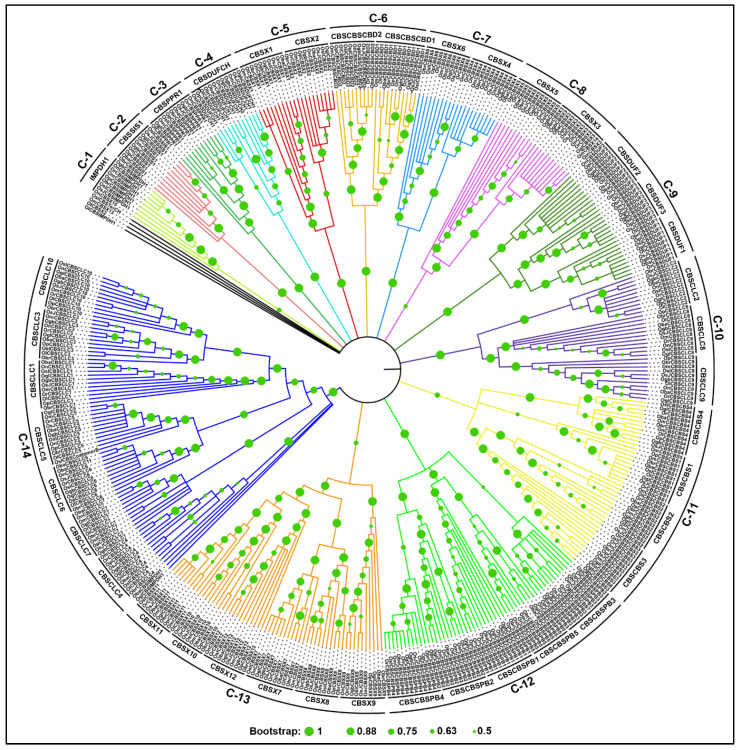
Phylogenetic tree depicting the relationship among various CDCPs identified in the 11 genomes from 10 *Oryza* sp. The tree comprises of fourteen major clades (C-1 to C-14), and each clade is depicted in a distinct color. Green circles represent the bootstrap values (1000 replicates).

To determine the evolutionary relationship among CDCP members in the cultivated and wild species of rice, a phylogenetic analysis was performed based on protein sequence alignment. The phylogenetic tree distributed all the CDCPs from 11 *Oryza* genomes into 14 major clades (referred herein as C-1 to C-14). The orthologous CDCPs from different species clustered together in the same clade, except a few CDCPs, namely ObaCBSIMPDH1, OglCBSIMPDH, OlCBSIMPDH, OlCBSX2, OmCBSX13, OnCBSX15, and OgbCBSCBS7, which were found to cluster distantly from the rest of their respective members (Figure 3).

The proteins containing only a single pair of CBS domains (CBSX1 to CBSX12) clustered into four distinct clades, implying functional diversification among these members. The orthologs of CBSX1 and CBSX2 in different rice species formed sister groups (in C-5). Similarly, the orthologs of CBSX3 and CBSX5, and CBSX4 and CBSX6 clustered in C8 and C-7, respectively, indicating that the proteins in each cluster have descended from the common ancestor. The orthologs of the newly classified CBSX7 to CBSX12 clustered together in the clade C-13, suggesting that the updated classification provided in this analysis is consistent with the evolution of these CDCPs. Notably, the protein sequence length of the members of CBSX7 to CBSX12 is exceptionally longer than that of the remaining CBSX members. The CBSX14, identified only in *O. meridionalis*, was found to have 100% sequence identity with OmCBSX3 and clustered with the CBSX3 proteins. Conversely, the CBSX13 and CBSX15, present only in *O. meridionalis* and *O. nivara*, respectively, were observed to be clustered distantly from all other CBSX members. The orthologs of CBSCBS1 to CBSCBS4 containing only two pairs of CBS domains, including CBSCBS5 and CBSCBS6, that were identified only in *O. meridionalis* and *O. glaberrima*, respectively, clustered together in C-11, signifying the common ancestor of these members. Moreover, the sequence alignment showed 100% identity between OgbCBSCBS6 and OgbCBSCBS3. However, the CBSCBS7 present only in *O. glaberrima* clustered with CBSCBSPB3 members, which implies that OgbCBSCBS7 might be a CBSCBSPB member that has lost its PB1 domain during the course of evolution. The orthologs of CBSCBSCBD1 and CBSCBSCBD2 possessing an additional CBD, which was previously classified as CBSX8 and CBSCBS4, respectively, clustered together in C-6.

Our previous phylogenetic study on CBSCLCs from various plant species clustered these CDCP members into two distinct groups: one consisting of a majority of these members, while others consisting of few members with higher identity to prokaryotic CLCs [18]. Likewise, in the present study, we observed the orthologs of CBSCLC1, CBSCLC3, CBSCLC4, CBSCLC5, CBSCLC6, CBSCLC7, and CBSCLC10 to jointly form a major CBSCLC clade (C-14) in *Oryza* species. On the other hand, the orthologs of CBSCLC2, CBSCLC8, and CBSCLC9 together formed a minor CBSCLC clade (C-10), and these proteins appeared to be more identical to prokaryotic CLCs (data not shown). This suggests that the two groups of CLCs have arisen from different ancestors. The C-14 cluster of CBSCLCs also included CBSIMPDH1 from *O. barthii* and *O. glumaepatula*, implying their distant relationship. However, the CBSIMPDHs from the rest of the species clustered independently into C-1. The orthologs of CBSCBSPB and CBSDUF family members clustered independently into C12 and C-9, respectively. Likewise, the orthologs of CBSSIS1, CBSPPR, and CBSDUFCH1 formed their distinct clades, C-2, C-3, and C-4, respectively.

Among the newly identified CDCPs in this study that possess additional hetero-domain, CBSCoatomerE present specifically in *O. sativa* subsp. *indica* is clustered with the orthologs of CBSCLC5 (C-14), which also include CBSCLC5 ortholog from *O. longistaminata* possessing additional CoatomerE domain. The CBSTerCH, present only in *O. sativa* subsp. *indica*, clustered distantly from the rest of the CDCPs. The sequence alignment showed 100% identity between the newly identified ObaCBSCLC11 and the previously known ObaCBSCLC6. Similarly, 100% identity was also observed between OgbDUFCH2 and OgbDUFCH1. Accordingly, these proteins clustered together in their respective sub-clades.

When we analyzed the branching pattern of CDCPs from 11 different genomes of *Oryza* species in each sub-clade and clade, we observed no consistent evolutionary relationship pattern among these *Oryza* species. Such inconsistency in the phylogenetic relationship among *Oryza* species with AA genomes has also been noted previously [1]. Nevertheless, we observed *O. brachyantha* (FF) CDCPs as early divergent or distant members in the majority of the clades, followed by *O. punctata* (BB) (Figure 3). Among the species with the AA genome, *O. longistaminata*, which has been regarded as the most ancestral species [30,31,32], appeared to be evolutionarily distant from the rest in most of the sub-clades or clades. Similarly, *O. brachyantha* has also been perceived to be distant from the rest of the species [33]. *O. longistaminata* is known to possess unique morphological features, such as self-incompatibility, rhizomatous, and the presence of distinct ligule, making it different from the rest of the AA genome species of *Oryza.* Moreover, *O. longistaminata* shows higher heterozygosity and a greater percentage of the presence of transposable elements (TEs) [34], indicating the accumulation of greater genomic variations from the rest of the species. TEs are known to comprise a major portion of plant genetic material and these potential endogenous mutation-causing agents have a significant role in the evolution of their respective host species [35]. TEs can bring about changes ranging from loss of function of any gene to complete reprogramming of the regulatory circuitry. Additionally, it has also been reported that TEs tend to be selectively removed from gene regions in the case of cultivated rice, and if present, they are more likely to occur in the intronic regions suggesting that cultivated rice species possess similar TE arrangements in their respective genomes [36].

In the case of the cultivated species, *O. sativa* and *O. glaberrima*, the orthologous genes associated with their domestication were reported to have undergone convergent, yet independent selection, and they share a high syntenic relationship [37]. In the present study, we also observed a closer relation between *O. sativa* subsp. *japonica* and *O. glaberrima*. The diversification in the *Oryza* species has occurred within a narrow time scale of about 15 million years. As such, analyzing the phylogenetic relationship for any multigene family proteins in domesticated and wild species would facilitate a better understanding of the evolution of the *Oryza* species.

### 2.3. Gene Structural Organization and Protein Motif Analysis of Different CDCPs

**Figure 4:**
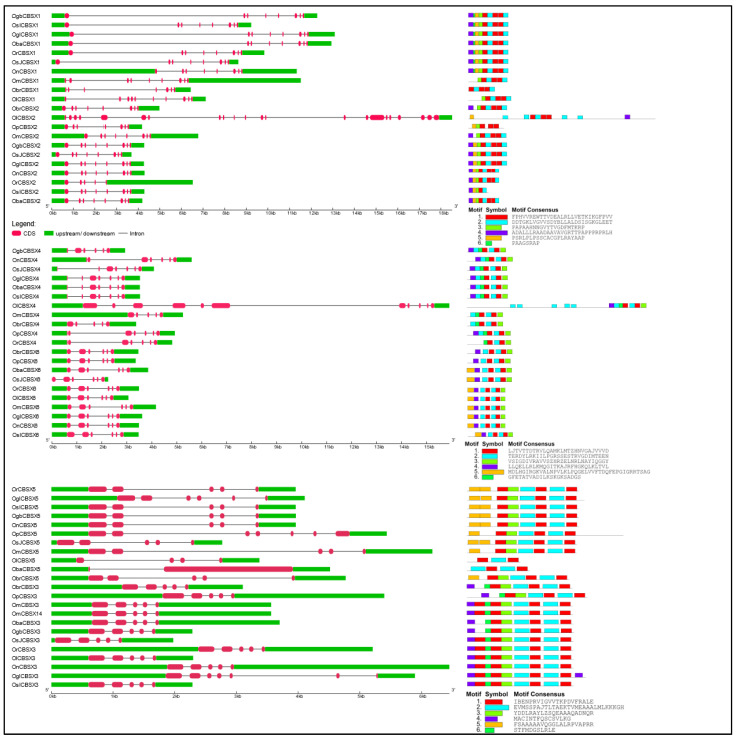
Schematic representation of the conservation of gene structure (left panel) and protein motifs (right panel) in the members of CDCPs containing only a single pair of CBS domains (CBSX1–CBSX6; excluding CBSX7–CBSX12, which are presented in Figure 5a) from different *Oryza* species. The length of UTR, exon, and intron has been depicted in proportion to the actual sizes, which is also indicated using a scale at the bottom. The order of different CDCPs is kept as per their phylogenetic relationship. The conserved motifs are predicted using the MEME suite.

**Figure 5:**
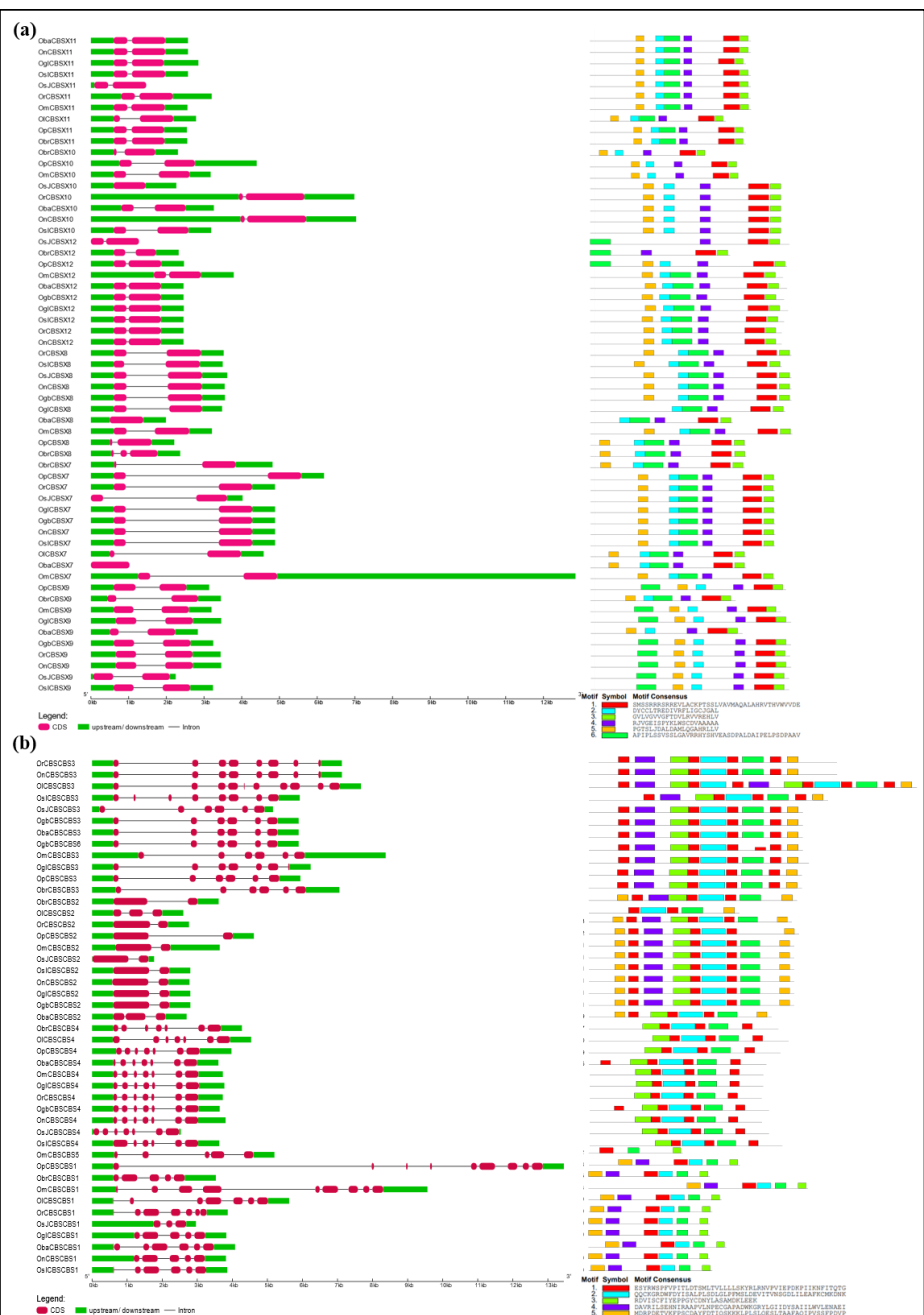
Schematic representation of the conservation of gene structure (left panel) and protein motifs (right panel) in CDCPs containing (**a**) only a single CBS domain containing proteins (CBSX7–CBSX12) and (**b**) only two pairs of CBS domains, from different *Oryza* species. The length of UTR, exon, and intron has been depicted in proportion to the actual sizes which is also indicated using a scale at the bottom. The order of different CDCPs is kept as per their phylogenetic relationship. The conserved motifs are predicted through the MEME suite.

**Figure 6:**
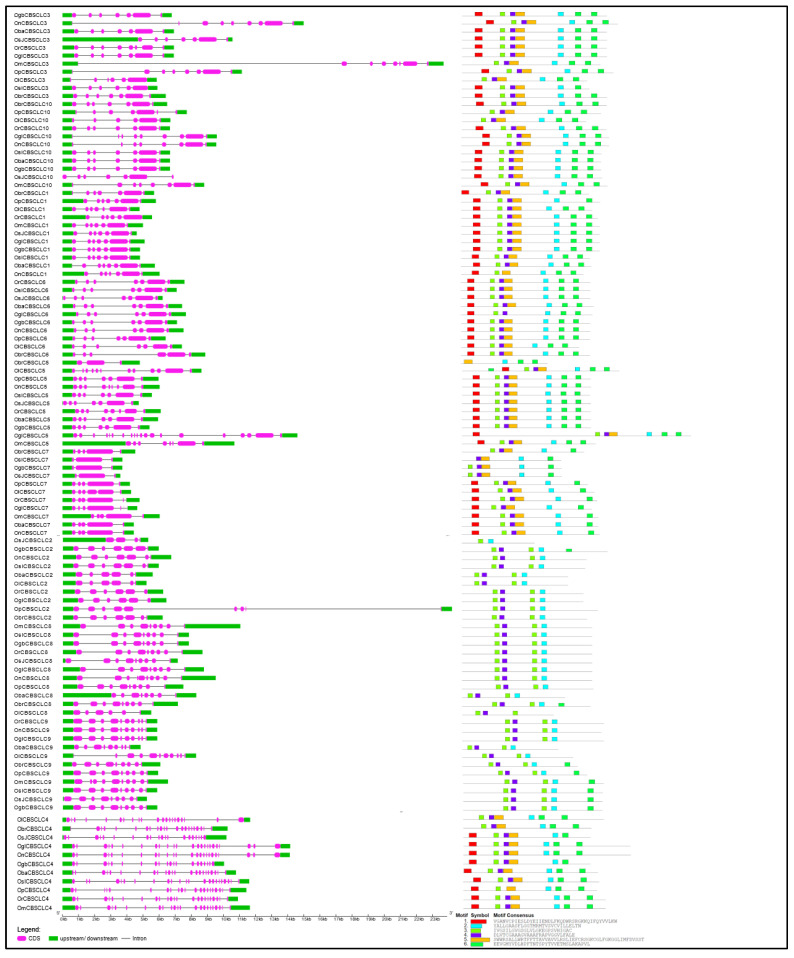
Schematic representation of the conservation of gene structure (left panel) and protein motifs (right panel) in CBSCLC members of the CDCP family from different *Oryza* species. Much similitude can be observed in terms of the genetic organization of different orthologs harboring the chloride channel domain. The length of UTR, exon, and intron has been depicted in proportion to the sequence lengths as indicated by the scale at the bottom. The order of the different CDCPs is kept as per their phylogenetic relationship. The conserved motifs are predicted through the MEME suite.

**Figure 7:**
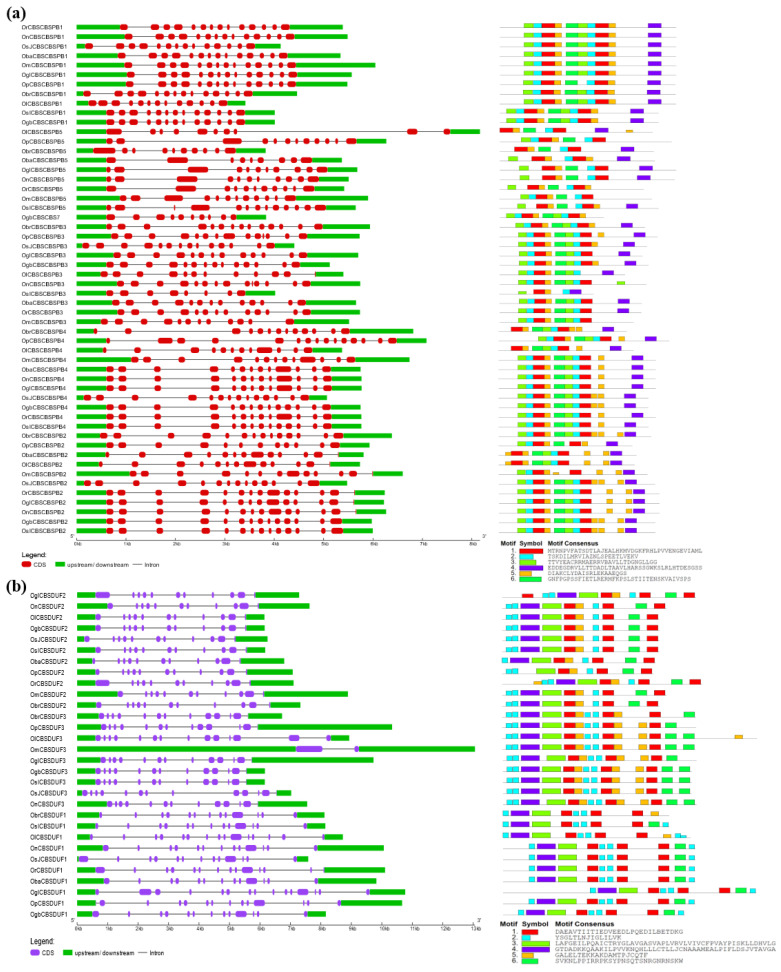
Schematic representation of the conservation of gene structure and protein motifs in CBSCBSPB and CBSDUF members of the CDCP family from different *Oryza* species. The length of UTR, exon, and intron are represented in correspondence to their respective sequence sizes. The different CDCPs are clustered according to their phylogenetic relationship. The conserved motifs are predicted using the MEME suite.

**Figure 8:**
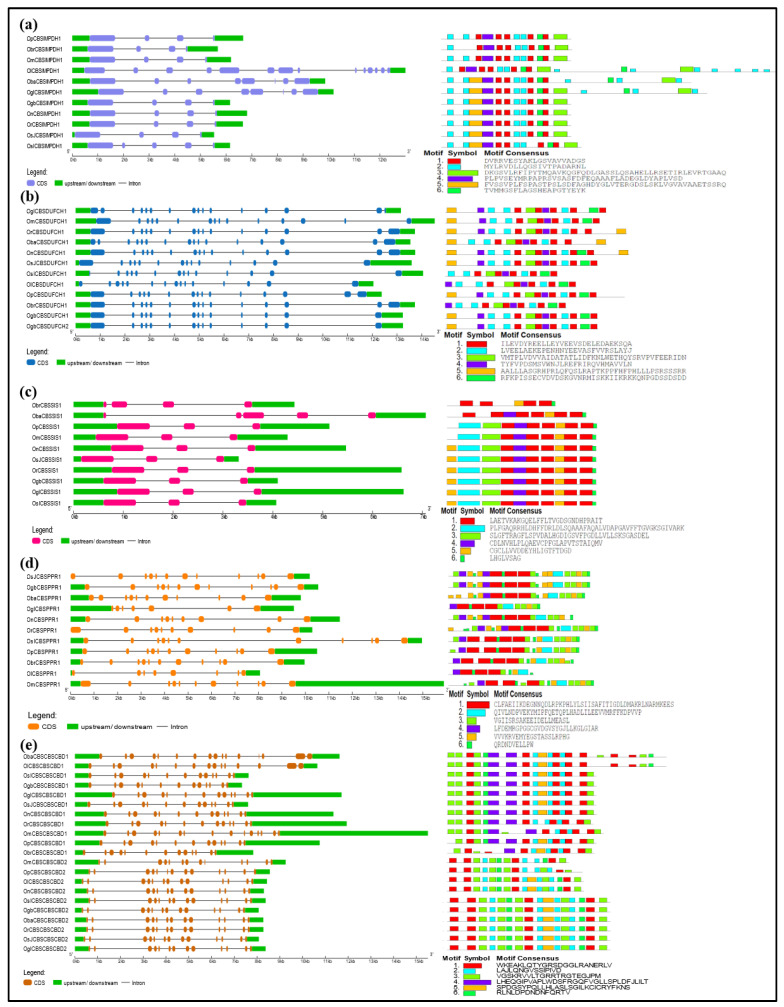
Schematic representation of the conservation of gene structure and protein motifs in (**a**) CBSIMPDH, (**b**) CBSDUFCH, (**c**) CBSSIS (**d**) CBSPPR, and (**e**) CBSCBSCBD members of CDCP family from different *Oryza* species. The respective length of UTR, exon, and intron for each ortholog are well in proportion to actual sequence lengths. The order of the different CDCPs is kept by their phylogenetic relationship. The conserved motifs are predicted through the MEME suite.

Following the phylogenetic analysis of the CDCPs in different *Oryza* species, we analyzed their gene structure as well the conservation of different protein motifs, to gain insight into their molecular diversity (Figure 4, Figure 5, Figure 6, Figure 7 and Figure 8). Generally, it has been found that a loss or gain of intron leads to structural complexity which functions as an important evolutionary force in the case of large protein families [38]. In the case of plants, genes with higher expression levels tend to have longer introns and untranslated regions in comparison to the low expressing ones [39]. In our analysis, we observed the orthologs of each CDCP from different *Oryza* species to exhibit high conservation in both gene structure and protein motifs. Corresponding to the phylogenetic study, the variations in gene structure and protein motif conservation were majorly observed in the CDCP members from *O. longistaminata*, followed by the ones from *O. brachyantha*, suggesting these two species as the distant ancestors, as reported previously [30,33].

In the case of CDCPs with only CBS domains, CBSX2 and CBSX4 from *O. longistaminata* were found to have longer sequences with additional motifs, while a large fraction of protein was found to be missing in CBSX5 from *O. longistaminata* and *O. barthii* (Figure 4). The CBSCBS3 from *O. longistaminata* also exhibited longer protein size with the presence of additional motifs, while a large region was missing in its other CDCPs, namely, OlCBSCBS2 and OlCBSX11. The CBSX8 orthologs from *O. brachyantha*, *O. punctata*, and *O. barthii* exhibited differences in both the gene structure and motifs arrangement. The CBSX10 and CBSX12 orthologs from *O. brachyantha* were observed to have lost a few motifs, while CBSX9 from both *O. brachyantha* and *O. barthii* showed loss of two motifs. Although OmCBSCBS5 was clustered with the members of CBSCBS1, it showed differences in both gene structure and motifs from CBSCBS1 orthologs (Figure 5). In CBSCBSCBD1 members, additional motifs were observed in orthologs from *O. longistaminata* and *O. barthii* (Figure 5).

In the case of CBSCLC sub-family, as CBSCLC2, CBSCLC8, and CBSCLC9 were identified to form distinct clades from the rest of the members in the phylogenetic tree, these proteins also exhibited different motif patterns from the remaining CLCs. A large region was observed to be missing in CBSCLC2 from *O. sativa* subsp. *japonica* and in CBSCLC5 from *O. brachyantha*, while CBSCLC5 from *O. glumaepatula* showed relatively longer gene and protein sequences (Figure 6).

Among CBSCBSPB family members, CBSCBSPB5 orthologs from *O. longistaminata* and *O. brachyantha* were found to differ from that of other species, mainly in gene structure. The CBSCBSPB4 from these two species also exhibited loss of a few protein motifs. Similarly, CBSCBSPB2 from *O. longistaminata*, *O. barthii*, *O. punctata*, and *O. meridionalis*, and CBSCBSPB3 from *O. sativa* subsp. *indica*, showed loss of a few motifs (Figure 7). In the CBSDUF family, all the members were found to have higher conservation in gene structure as well as in protein motif patterns (Figure 7).

By the phylogenetic analysis, the CBSIMPDH1 orthologs from *O. longistaminata*, *O. barthii*, and *O. glumaepatula* differed in gene structure and protein length (with additional motifs). Among CBSDUFCH1, its orthologs are from *O. longistaminata*, *O. brachyantha*, *O. barthii*, *O. rufipogon*, and *O. sativa* subsp. *indica* showed a lack of one or few motifs. In CBSSIS1, a shorter protein length with a lack of few motifs was observed in its orthologs from *O. brachyantha* and *O. barthii*. In CBSPPR1, the shorter protein size was observed in its orthologs from *O. longistaminata* and *O. glumaepatula* (Figure 8).

Although the orthologs often possess high sequence similarity at the protein level, there may have differences acquired in the length of 5′ and 3′ UTR at the gene level, which are known to subsequently provide functional specificity [40]. Additionally, in the case of plants, the developmental transitions, as well as response towards environmental adversities, are modulated through transcriptional reprogramming, which is substantially coordinated by the UTRs [40]. During evolution, with an increase in genome sizes, the UTRs have been found to lengthen, particularly the 3′ UTR [41]. Moreover, some 5′ UTRs and 3′ UTRs harbor certain regulatory sequences that may act on the stability and localization of mRNA, as well as its translational efficiency [42,43,44]. The efficient translation facilitating 5′ UTRs have been observed to be short, with less GC content and secondary structures. On the contrary, longer and highly structured 5′ UTRs are more often than not associated with the genes regulating highly specific developmental processes, particularly in a tissue-specific manner [45]. In congruency with the above statements, we observed marked differences in the length of UTRs between the orthologs of CDCPs belonging to different subfamilies. Unprecedently long 5′ UTRs were observed in the case of all the orthologs of *CBSX3* and *CBSX5*. Longer 5′ UTRs were also observed in *OnCBSX1*, *OmCBSX4*, *OrCBSCLC7*, *OnCBSCLC7*, *OsJCBSCLC2*, *OsJCBSCLC3*, *OmCBSCLC5*, *ObaCBSCLC8*, and *OmCBSDUF3* among their respective orthologs from other *Oryza* species, supporting a length-dependent functional precision as reported by Srivastava et al. [40]. Interestingly, only in *O. sativa* subsp. *japonica*, we noticed that some CDCP members were devoid of either 5′UTR or 3′ UTR, or both, such as in *OsJCBSCLC10*, *ObaCBSX7*, and *OsJCBSX12*, suggesting a complex and alternative post-translational regulatory mechanism for such genes [44].

### 2.4. Gene Duplication and Synteny Analysis in Various Oryza Species

**Figure 9:**
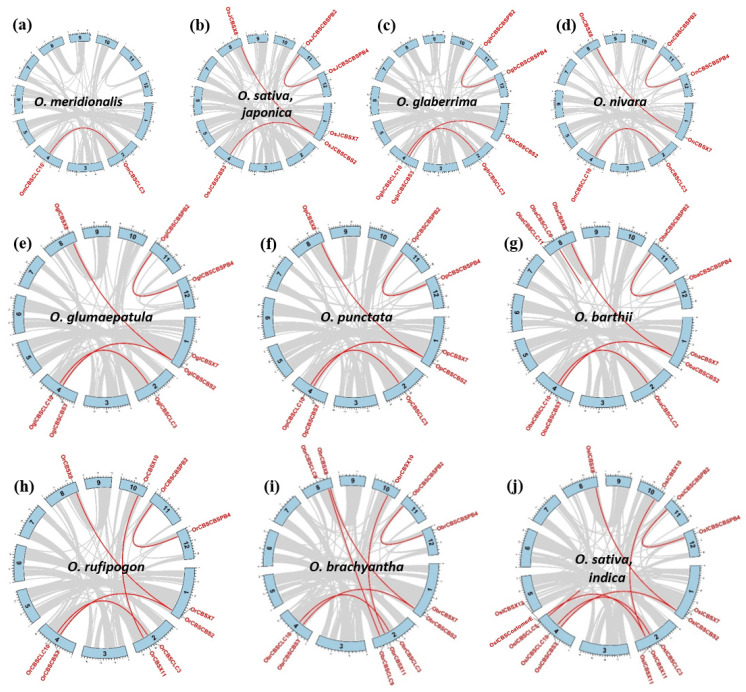
Gene duplication analysis of CDCP genes in different *Oryza* species. The duplication analysis was carried out in 10 genomes of 9 *Oryza* species, namely (**a**) *O. meridionalis* (**b**) *O. sativa* subsp. *japonica* (**c**) *O. glaberrima* (**d**) *O. nivara* (**e**) *O. glumaepatula* (**f**) *O. punctata* (**g**) *O. barthii* (**h**) *O. rufipogon* (**i**) *O. brachyantha*, and (**j**) *O. sativa* subsp. *indica*. The chromosome number is shown in the middle of each chromosome and the genomic location for each gene has been marked on the respective chromosomes. The grey lines in the background represent the collinear blocks within each genome. The red-colored lines highlight the duplication events present. The gene duplication analysis was carried out using the MCScanX toolkit.

Gene duplication events and their subsequent retention contribute to the evolution of novel functions and stress adaptation in plants [46,47,48,49]. Using the MCScanX algorithm, which considers both homology and genomic distribution to evaluate the collinearity and synteny, we detected duplications in the CDCP genes in all 10 rice genomes, which ranged from 1–6 in number (Figure 9; Table 2). Since the annotation for *O. longistaminata* is available only up to scaffold level, it was excluded from the gene duplication analysis. Our analysis suggested that the whole genome or segmental duplication events have led to the expansion of the CDCP gene family in all the *Oryza* species.

To understand the selection pressure on the duplicated CDCP genes, the non-synonymous (Ka) and synonymous substitutions (Ks), and also the Ka/Ks ratios were calculated for the duplicated gene pairs. The value of Ka/Ks = 1 suggests that the genes have undergone a neutral selection, while <1 and >1 values suggest negative and positive selection, respectively [50]. We observed Ka/Ks values < 1 for all the genes encoding CDCPs, indicating that these genes in all the *Oryza* species have experienced negative or purifying selection pressure during evolution (Table 2). Prevalence of negative selection indicates optimization of genetic structures through long-term evolutionary processes such that any mutational change in genes leads to a reduction in biological fitness. Negative selection, thus, maintains the fixation of genetic characters in a population by removing deleterious mutations [51]. Additionally, in the paralogous gene pairs encoding the CDCPs, the purifying selection pressure appears to act more strongly on the non-synonymous mutations than the silent mutations. Thus, preserving the functional properties of CDCPs in all the 10 *Oryza* genomes evaluated even after duplication, as a further adaptive advantage by a mutation on a non-synonymous site might be an unlikely event. Several other gene families in *Oryza* sp., such as WRKY [52], ALOG domain [53], DUF-221 domain containing gene family [54], F-box, and NB-ARC gene families [55], have also been found to be shaped majorly by negative selection. The gene duplication has contributed to the expansion of the gene family through segmental or whole-genome duplication in rice species [56]. In a previous study on rice, duplicated blocks resulting from a whole-genome duplication event were found to cover about 60% of the genome [57]. The large-scale duplication of the rice genome was also reported by Wang et al. [58] in *Oryza sativa* subsp. *indica*.

To study synteny relationships of the CDCP genes from the cultivated species, *O. sativa* subsp. *japonica*, with the genes from other rice genomes, orthologous genes were identified between genomes of *O. sativa japonica* and each of the other rice species using MCScan Toolkit (Supplementary Figure S1). Most of the orthologous gene pairs were collinear, manifesting conservation of synteny blocks. A total of six, seven, six, seven, seven, five, nine, six, and seven non-collinear orthologous pairs were found between *O. sativa* subsp. *japonica* CDCP genes and that of *O. barthii*, *O. brachyantha*, *O. glaberrima*, *O. glumaepatula*, *O. sativa* subsp. *indica*, *O. meridionalis*, *O. nivara*, *O. punctata*, and O. *rufipogon*, respectively. Few of the CDCP genes have two orthologous genes—one collinear and another non-collinear—which indicates that the genes in collinear and non-collinear positions might have evolved from common ancestors. For instance, each of *OsJCBSCBSPB2* and *OsJCBSCBSPB4* genes showed orthology with both *CBSCBSPB2* and *CBSCBSPB4* of *O. barthii*, *O. brachyantha*, *O. glaberrima*, *O. glumaepatula*, *O. sativa* subsp. *indica*, *O. nivara*, *O. punctata*, and *O. rufipogon.*

### 2.5. Analysis of Cis-Elements in the Promoter Sequence of Genes Encoding CDCPs

**Figure 10:**
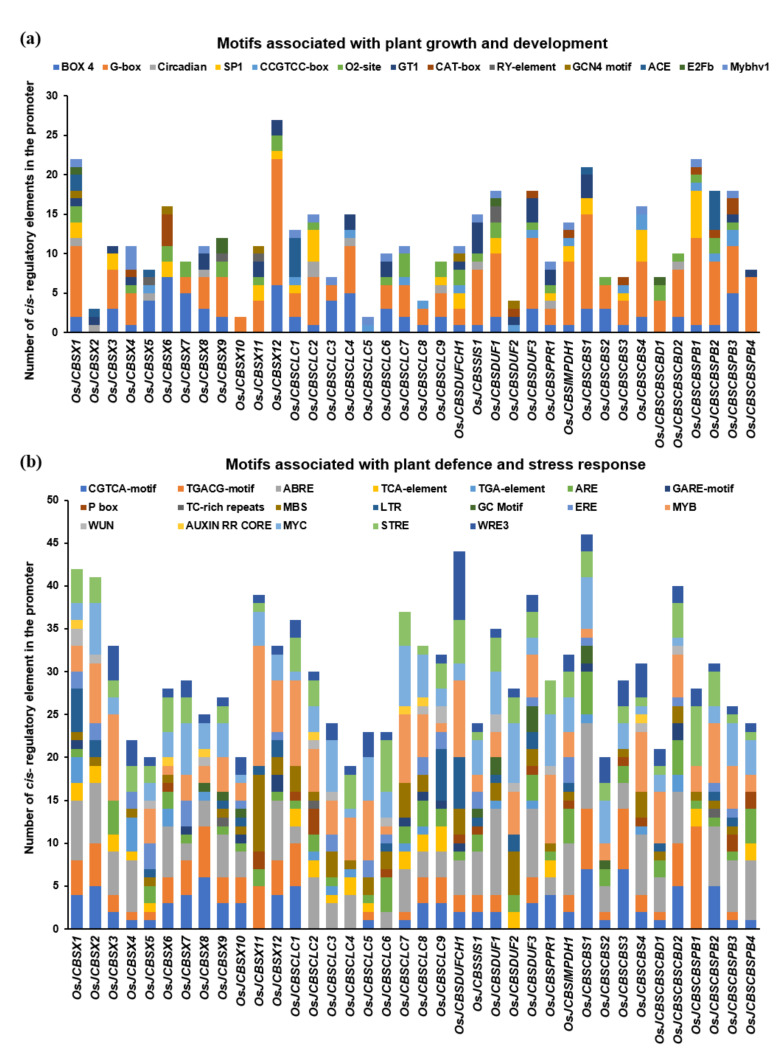
Analysis of the *cis-*regulatory elements present in the 2 kb upstream promoter region of different CDCP genes from *O. sativa* subsp. *japonica*. (**a**) Motifs related to plant growth and development and (**b**) motifs related to plant defense and stress response. Each colored box and its length in the bar represent a specific motif and its frequency of occurrence in the promoter, respectively.

To delve into the evolution and functional divergence of the CDCPs, the 2 kb upstream promoter regions of all the genes encoding CDCPs (except *OsJCBSCLC10*, whose chromosomal location is not annotated in the genome) from *O. sativa* subsp. *japonica* were analyzed using the PlantCARE tool. Different *cis*-acting regulatory elements pertaining to both plant development and stress responses were predicted to be present in their promoter sequences (Figure 10; Supplementary Table S1). The motifs related to plant growth and development included Box 4, G-Box, SP1 and GT1 (light-responsive), zein metabolism and CAT motif (meristem specific expression), RY element (seed-specific), and GCN4 motif (endosperm specific). Additionally, the auxin-responsive AuxRR-core and TGA-element, the gibberellin-responsive GARE motif and P-box motifs, and the salicylic acid-responsive TCA-elements were also identified. The light-responsive Box 4 and G-box *cis*-regulatory elements were found abundantly in the promoter sequences of most of the genes encoding CDCPs. Specifically, the G-box is a hexameric DNA motif associated with the transcription induction of genes in response to light as well as senescence in the leaf [59,60]. This regulatory element was found in the promoters of all the genes encoding CDCPs, except for *OsJCBSX2*, *OsJCBSX5*, *OsJCBSX6*, *OsJCBSCLC5*, and *OsJCBSDUF2* genes. The Sp1 light-responsive element was predominantly found in the promoter sequences of the *OsJCBSX* subfamily, indicating their photoperiod-dependent mode of functioning. Additionally, the presence of other light-responsive elements in the promoters of CDCP genes suggests circadian control as well as a putative role in photomorphogenesis.

It may be noted that the promoter of *CBSX1*, *CBSX9*, *OsJCBSCBSCBD1*, and *CBSDUF1* contained an E2Fb transcription factor binding motif. In *Arabidopsis*, the E2Fb in complex with the Dimerization Partner (DP) has been reported to be involved in the DNA repair process during cell division [61]. The E2Fb has been reported to stimulate cell division [62] and is important during the post-mitotic state to resolve organ size [63]. Additionally, the E2Fa and E2Fb transcription factors have also been reported to be induced by a protein kinase Target of Rapamycin (TOR), which in turn is regulated through a small GTPase ROP2 protein in light-dependent as well as in auxin-dependent manner [64]. This suggests the putative role of *OsJCBSCBSCBD1* in cell division and DNA repair.

The promoter sequences of CDCP genes were also found to contain a higher frequency of stress-responsive *cis*-regulatory elements, such as ARE (anaerobic responsive element), MBS (MYB transcription factor binding site), MYB (stress-responsive), ABRE (ABA-Abscisic acid-responsive element), LTR (low temperature-responsive), TC-motif (stress responsiveness), CGTCA and TGACG motifs (Me-JA responsive), GC motif (Anoxia), STRE (stress-responsive), WRE3 (heat-responsive), WUN (wounding), and ERE (ethylene-responsive). The ARE motifs have been established as key motifs in the promoter regions of anaerobically induced proteins (ANPs) [65]. Except for *OsCBSX2*, *OsCBSX4*, *OsCBSX8*, *OsCBSCBS4*, *OsCBSCLC4*, and *OsCBSDUF1*, the promoters of all other CDCP genes were found to contain at least one ARE element. The ability to cope with varying degrees of oxygen limitations occurs through the induction of genes encoding for enzymes participating in carbohydrate metabolism, fermentation as well as survival pathways [66]. Although CDCPs do not have a defined role in the case of oxygen stress to date, the presence of AREs suggests that these proteins might have important functions under anaerobic growth conditions.

ABRE is another major *cis*-acting regulatory element well-known to have an indispensable role in acclimation to abiotic stresses [67]. It is also known to regulate seed maturation and dormancy [68]. We observed this element to be present in different genes encoding CDCPs, except *OsJCBSX5*, *OsJCBSX11*, *OsJCBSCLC1*, *OsJCBSCLC5*, *OsJCBSCLC6*, *OsJCBSDUF2*, *OsJCBSCBSPB1*, and *OsJCBSCBSPB2*. In the case of the transcriptional activation of the pathogen-related proteins, salicylic acid (SA) and jasmonic acid, along with their methyl ester, are the prime signals known in plants [69]. Two methyl jasmonate responsive motifs- CGTCA and TGACG, were identified in all the CDCP genes, except for *OsJCBSDUF2* and three CBSCLC subfamily genes, namely *OsJCBSCLC2*, *OsJCBSCLC3*, and *OsJCBSCLC4.* Moreover, the SA-responsive TCA element was found in the promoter sequences of many CDCP genes, including *OsJCBSX2–OsJCBSX4*, all *CBSCLCs* (except *OsJCBSCLC5* and *OsJCBSCLC6*), *OsJCBSPPR1*, and two members of the *CBSCBSPB* subfamily (*OsJCBSCBSPB1* and *OsJCBSCBSPB4*).

A maize *Viviparous 1* gene (*VP1*), a homolog of *Arabidopsis ABI3*, is known to carry out two important functions, including embryo maturation and germination repression [70]. The *VP1* is also known to alter the ABA-responsive gene expression including the maturation associated genes [71,72,73] by directly binding to the RY *cis*-regulatory element [74]. We observed the presence of the RY element in three members of the *CBSX* family, namely *OsJCBSX5*, *OsJCBSX9*, and *OsJCBSX11*, as well as in *OsJCBSDUF1*. The presence of the RY element along with ABRE in the case of *OsJCBSX9* and *OsJCBSDUF1* suggests that these genes might be involved in seed maturation and/or seed dormancy as well in imparting stress tolerance.

Altogether, the promoter sequence analysis of genes encoding CDCPs from *Oryza sativa* subsp. *japonica* indicates their involvement in diverse growth and developmental processes as well as in stress responses.

### 2.6. Developmental and Stress-Responsive Regulation of Genes Encoding CDCPs

**Figure 11:**
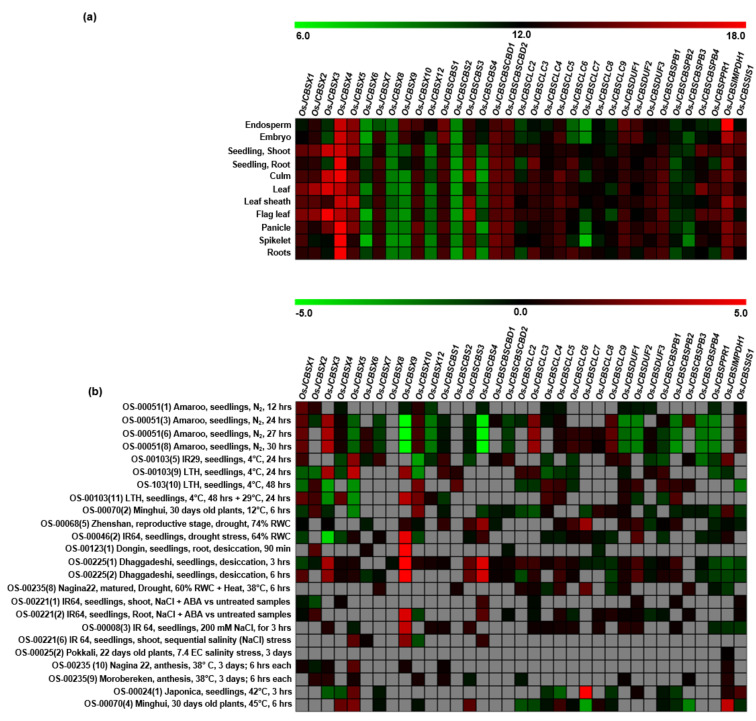
The in silico expression analysis of different CDCP genes in *O. sativa* subsp. *japonica* in (**a**) different tissues during the course of development and (**b**) under different stress conditions. Expression data were retrieved from the publicly available Affymetrix as well as RNA-seq datasets from the Genevestigator. The data in (**a**) are presented as linear expression value as used in the Genevestigator, and the data in (**b**) are presented as Log2 of fold change. The expression data in (**b**) are filtered with *p*-value < 0.05, while the data with *p*-value > 0.05 are left empty as grey boxes. The data have been depicted as a heatmap created using the MeV tool. The color scale above each heatmap shows the level of expression.

To understand the anatomical, developmental, and stress-responsive expression of different CDCP genes in the cultivated *O. sativa* subsp. *japonica*, the gene expression profiles were retrieved from the GENEVESTIGATOR database. For a comprehensive analysis, datasets from both Affymetrix and RNA-seq experiments were taken into consideration. However, it should be noted that the expression data for *OsJCBSX11*, *OsJCBSCLC1*, *OsJCBSCLC10*, and *OsJCBSDUFCH1* were unavailable in the database; hence, they could not be analyzed in the present study. We found marked variations between transcripts of different genes encoding CDCPs in various tissues (Figure 11a). The *OsJCBSX4*, *OsJCBSCBS1*, *OsJCBSCBSCBD1*, *OsJCBSDUF1*, and *OsJCBSIMPDH1* exhibited consistently higher expression levels in all the tissues, suggesting their prominent role in diverse growth and development processes. Though not many studies have been conducted on these CDCPs, Zafar et al. [12] recently reported the role of OsCBSDUF1 (they termed it as Degenerated Panicle and Partial Sterility (DPS1)) in seed setting via regulation of ROS homeostasis. This finding strengthens our observation that these CDCPs have a crucial role in plant growth and developmental processes. In another instance, we found *OsCBSX9* to have higher expression levels in the endosperm, embryo, and seedling root, indicating its role in seed storage or germination. Likewise, *OsCBSCBS4* also showed elevated expression only in the endosperm and embryo.

Duplication events are known to increase the expression diversity, thereby enabling specialized or distinct evolutionary patterns for the development of an organism [75]. The duplicated pair of *OsCBSCBS2* and *OsCBSCBS3* showed such a variation in terms of their expression levels. *OsCBSCBS2* maintained relatively lower but constant expression levels in all the tissues, whereas *OsCBSCBS3* showed higher expression in a tissue-specific manner. This is in correlation with the fact that plant genes involved in signal transduction, transcriptional regulation, and stress response favor to have paralogs [48]. More often, paralogs have a propensity to bifurcate the ancestral functions in a way that both the gene copies become vital for the organism and are, therefore, retained by the system through the dynamic process of evolution [76,77]. Alternatively, one duplicate copy of the gene might give rise to advanced expression patterns or new functions (neo-functionalization) [78,79]. The duplicated pair of *OsCBSCBSPB2* and *OsCBSCBSPB4* showed such a variation with the latter showing higher fold expression in the case of seedling root, leaf, and flag leaf tissues in comparison to its duplicate copy, suggesting neo-functionalization. Hoffman and Palmgren [80] previously attempted to establish a link between the retention of paralogous genes and their expression. They observed a correlation between the Ka/Ks ratio and the expression of the paralogous genes in *Arabidopsis*. We also found similar observations in two sets of paralogs from *O. sativa* subsp. *japonica*, *OsCBSCBS2* and *OsCBSCBS3*, and *OsCBSCBSPB2* and *OsCBSCBSPB4*. Although both these paralogs showed differences in tissue specificity, which was found to be more in the case of *OsCBSCBS2* and *OsCBSCBS3*, as indicated by its lower Ka/Ks ratio in comparison to *OsCBSCBSPB2* and *OsCBSCBSPB4* gene pair, suggesting a difference in the intensity of the purifying selection acting during evolution.

Furthermore, to gain insight into the function and relevance of CDCPs in plant survival under abiotic stresses, their gene expression data under various abiotic stresses, namely, anoxia, cold, drought, salinity, and heat stresses, were also retrieved and analyzed (Figure 11b). We observed higher expression levels of *OsCBSX1*, *OsCBSX3*, *OsCBSCLC3*, *OsCBSCLC9*, *OsCBSSIS1*, and *OsCBSX10* in anoxic conditions at variable time periods ranging from 12–30 hours. Similarly, different CDCP genes such as *OsCBSX2*, *OsCBSX9*, *OsCBSCLC4*, *OsCBSCLC5*, and *OsCBSDUF1* exhibited higher expression levels under cold conditions. Additionally, significantly higher expression levels were observed in the case of *OsCBSX9* and *OsCBSCBS4* under drought as well as salinity stress conditions. Apart from these genes, many other CDCPs such as *OsCBSX4*, *OsCBSX5*, *OsCBSDUF2*, and many *OsCBSCLCs* exhibited relatively higher expression under heat stress conditions. This observation correlated well with the role of *OsCBSX4* in tolerance against multiple stress responses [5]. Additionally, overexpression of Soybean genes, namely *GmCBS21* and *GmCBSDUF3*, have been reported to enhance tolerance to low nitrogen and multiple abiotic stresses, respectively [14,15]. It should also be noted that substantial difference in expression levels between the gene duplicate pairs—*OsCBSCBS2* and *OsCBSCBS3*; *OsCBSCBSPB2* and *OsCBSCBSPB4*—could also be seen suggesting divergence in their functions.

The difference in expression levels in the case of different tissues could be linked with the presence of different *cis*-regulatory elements. In the case of cotton, it has been reported that about 40% of homologs formed from a whole-genome duplication event have transcriptional divergence primarily because of the difference in *cis*-regulatory elements [81]. On comparing the same, we observed a marked difference in the *cis*-regulatory elements present in the promoters of the duplicated pairs—*OsCBSCBS2/OsCBSCBS3*, *OsCBSCLC3/OsCBSCLC9*, and *OsCBSCBSPB2/OsCBSCBSPB4* (Figure 10). Additionally, it has been reported that genes having similar patterns of expression possess alike motifs in their respective promoter regions [82]. For example, the drought stress responsiveness observed in the case of *OsCBSX9*, *OsCBSX12*, and *OsCBSCBS4* could be attributed to the presence of a MYB binding site for drought inducibility (MBS) in their promoter regions. Likewise, higher fold expression levels in response to hypoxia stress were observed in the case of *OsCBSX1*, *OsCBSX3*, *OsCBSCLC3*, *OsCBSCLC9*, *OsCBSSIS1*, and *OsCBSX10* converge at the point of presence of ARE in their respective promoter regions. Taken together, it can be concluded that the *cis*-elements play a vital role in shaping the expression divergence of genes during the course of evolution.

**Figure 12:**
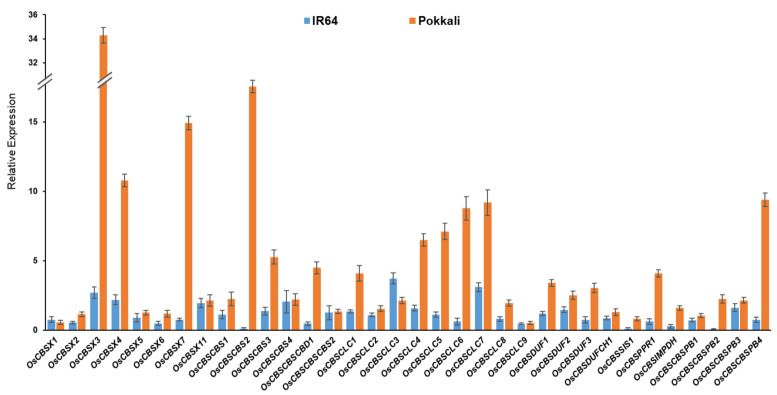
The qRT-PCR based expression analysis of different CDCP genes in the shoot tissues of the seedlings of IR64 and Pokkali in response to salinity treatment (200 mM NaCl). The expression data are expressed as relative fold change against the untreated control samples. The experiment was repeated twice with three replicates in each case. Error bar represents the standard deviation (*n* = 6).

We further analyzed the expression of selected CDCP genes by qRT-PCR in two contrasting rice genotypes (*Oryza sativa* subsp. I*ndica*), namely, IR64 (salt-sensitive) and Pokkali (salt-tolerant), in response to salinity treatment (200 mM NaCl) in the shoot tissues at the seedling stage. Importantly, the expression of many members, namely, *OsCBSX3*, *OsCBSX4*, *OsCBSX7*, *OsCBSCBS2*, *OsCBSCBS3*, *OsCBSCBSCBD1*, *OsCBSCLC1*, *OsCBSCLC4-7*, *OsCBSPPR1*, and *OsCBSCBSPB4*, were highly induced in salt-tolerant Pokkali, while salt-sensitive IR64 rice did not manifest the induction of most of these genes (Figure 12). Such differential expression of the members of the CDCP genes implies their possible association with the salinity tolerance traits in the tolerant genotype.

## 3. Materials and Methods

### 3.1. Data Retrieval and Sequence Analysis

The HMM (Hidden Markov Model) profile of the CBS domain (PF00571) was retrieved from the Pfam database, (http://pfam.xfam.org/; EMBL-EBI, UK; accessed on 24 June 2021), which was used to identify the full-length protein sequences of CDCPs in the rice genome (*O. sativa* subsp. *japonica*) from MSU Rice Genome Annotation Project Database (http://rice.uga.edu; MSU, USA; accessed on 24 June 2021). The protein blast (blastp) search was performed using each rice CDCP sequence as a query in the Gramene database *(*https://www.gramene.org, accessed on 26 June 2021) for the identification of homologous sequences in *O. sativa* subsp. *indica* and nine other *Oryza* species, namely *O. barthi*, *O. brachyantha*, *O. glumaepatula*, *O. glaberrima*, *O. longistaminata*, *O. meridionalis*, *O. nivara*, *O. punctata*, and *O. rufipogon*. All the retrieved sequences were examined with the Pfam 34.0 tool (http://pfam.xfam.org/, accessed on 7 July 2021) for the presence of CBS and other hetero-domains, and the proteins were named according to Kushwaha et al. [4] with some changes in the previous classification system.

### 3.2. Phylogenetic Analysis

Multiple sequence alignment and the construction of a phylogenetic tree was performed using MEGA7.0 [83]. The sequences were aligned following MUSCLE method (default parameters) and the evolutionary tree was constructed following a Neighbor-joining method (1000 bootstrap replicates). The tree was visualized and annotated using iTol (https://itol.embl.de/upload.cgi, accessed on 24 July 2021).

### 3.3. Gene Structure and Motif Analysis

Structural visualization of the genes was performed using Gene Structure Display Server 2.0 (GSDS 2.0) (http://gsds.cbi.pku.edu.cn/; Peking University; accessed on 15 October 2021). The putative CDCP sequences were analyzed for the presence of conserved motifs using the Multiple Expectation Maximization for Motif Elicitation (MEME) program (http://meme-suite.org/, accessed on 20 July 2021) with the default parameters and the maximum number of motifs was set as 6.

### 3.4. Chromosomal Distribution, Duplication Analysis, and Synteny

Information of chromosome coordinates and annotations (.gff files) of the rice species were obtained from the plant Ensembl database (https://plants.ensembl.org/index.html, accessed on 7 July 2021). The full-length CDS and the protein sequences were also downloaded from the Ensembl database. To investigate gene duplication events of CDCPs within a species, the MCScanX toolkit [84] with its default parameters was employed using the output of blastp homology search within 10 rice species. To evaluate selection pressure between the paralogous genes pairs, the non-synonymous substitution rate (Ka), synonymous substitution rate (Ks), and the pairwise Ka/Ks ratios were estimated using TBtools [85]. TBtools was also used to generate Circos plots to visualize the paralogous pairs. The annotation for *O. longistaminata* was available only up to scaffold level; thus, it was excluded from the gene duplication analysis.

### 3.5. In Silico Promoter Analysis of CDCPs

The 2 kb upstream promoter sequences from the transcription start site of different CDCP genes were retrieved from *Oryza sativa* subsp. *japonica* using the RAP-DB database (https://rapdb.dna.affrc.go.jp/, accessed on 28 July 2021) and analyzed using the PlantCARE database (http://bioinformatics.psb.ugent.be/webtools/plantcare/html/, accessed on 28 July 2021) for the presence of different developmental-associated as well as stress-responsive *cis*-regulatory elements in these promoter sequences.

### 3.6. Developmental and Stress Mediated Expression Profiling of Different CDCPs in O. sativa

The expression profiles of different CDCP genes of *Oryza sativa* subsp. *japonica* under various abiotic stress conditions, such as heat, cold, salinity, anoxia, and drought, as well as in various tissue at different developmental stages, were retrieved from the publicly available Genevestigator database (https://genevestigator.com; NEBION, Switzerland; accessed on 22 July 2021). The data were presented in the form of heatmaps using MeV 4.9.0 tool [86].

For qRT-PCR based expression analysis, 10-day old seedlings of IR64 and Pokkali were treated with 200 mM of NaCl for 6 hr. Total RNA was isolated from the shoot tissues using TRIZOL reagent (ThermoFisher Scientific, Waltham, MA, USA), followed by cDNA preparation using RevertAid First Strand cDNA Synthesis Kit (ThermoFisher Scientific, Waltham, MA, USA) and qRT-PCR (Applied Biosystems 7500, Foster City, CA, USA). The eukaryotic *Elongation Factor-1α* (*eEF-1α*) was used as an internal control for normalization in the qRT-PCR [87]. The gene expression data were analyzed based on the comparative CT method [88].

## 4. Conclusions

The CBS domain containing proteins constitute a large superfamily with only a few members characterized functionally. In the present study, we explored the CDCP family in the genomes of six wild species of *Oryza* with diploid AA genome, namely, *O. longistaminata*, *O. rufipogon*, *O. glumaepatula*, *O. meridionalis*, *O. nivara*, and *O. barthii*, along with two domesticated species, namely *O. sativa* (subsp. *Japonica* and *indica*) and *O. glaberrima.* These eight species form a pioneer gene pool and can be crossed easily for breeding purposes. Other than these, two more species, *O. brachyantha* (FF genome) and *O. punctata* (BB genome), were also included in this study to identify the evolutionary relationships among these stress-responsive CDCP genes in different *Oryza* species. Through this comparative analysis, we identified three previously unreported hetero-domains associated with CBS domains in CDCPs, namely, TerCH, CoatomerE, and CBD. Additionally, we also identified other new members in previously known CDCP subfamilies. Their phylogenetic analysis suggested CDCPs possess high sequence conservation, as also indicated by their gene structure organization. The gene expression analysis revealed their differential expression under a single as well as multiple stresses, suggesting their involvement in various stress regulatory pathways. The expression of some members was also observed to be differential in the salt-tolerant and salt-sensitive rice genotypes in response to salinity. Altogether, this study provides novel insights into the classification, evolutionary conservations, and functional divergence of the members of the CDCP family across different *Oryza* species, which in the future can help researchers in pursuing functional characterization of these proteins. The stress-responsiveness of some members of CDCP genes noted in this study encourages their further study for improving stress tolerance in domesticated *Oryza* species.

## Figures and Tables

**Table 1:** New classification and the distribution of CDCPs in 11 genomes from 10 *Oryza* species. Note that the ‘old classification’ represents the previous nomenclature and classification of CDCPs from *O. sativa* subsp. *japonica* genome by Kushwaha et al. [4], which has been updated with a few changes based on updated protein domain prediction and the identification of new CDCP members. The blank space denotes the absence of the corresponding ortholog in the respective species and ‘-’ in the old classification column represents that the CDCP member was not previously identified.

Old Classification	New Classification	*O. sativa*, *japonica*	*O. barthii*	*O. brachyantha*	*O. glaberrima*	*O. rufipogon*	*O. punctata*	*O. nivara*	*O. meridionalis*	*O. longistaminata*	*O. sativa*, *indica*	*O. glumaepatula*
CBSX1	CBSX1	LOC_Os08g22149	OBART08G10130	OB08G19240	ORGLA08G0088800	ORUFI08G11440		ONIVA08G10830	OMERI08G08660	KN540332.1_FG002	BGIOSGA014003	OGLUM08G11030
CBSX2	CBSX2	LOC_Os09g02710	OBART09G00750	OB09G10450	ORGLA09G0006600	ORUFI09G00780	OPUNC09G00530	ONIVA09G00790	OMERI09G00790	KN538975.1_FGP001	BGIOSGA030223	OGLUM09G01050
CBSX3	CBSX3	LOC_Os02g57280	OBART02G37390	OB02G44090	ORGLA02G0326000	ORUFI02G38890	OPUNC02G34550	ONIVA02G40140	OMERI02G35160	KN539631.1_FGP002	BGIOSGA009298	OGLUM02G38520
CBSX4	CBSX4	LOC_Os03g52690	OBART03G33380	OB03G40470	ORGLA03G0303100	ORUFI03G34760	OPUNC03G30600	ONIVA01G07590	OMERI03G30610	KN539195.1_FGP002	BGIOSGA009847	OGLUM03G33040
CBSX5	CBSX5	LOC_Os04g05010	OBART04G01510	OB04G11410	ORGLA04G0012700	ORUFI04G01970	OPUNC04G01560	ONIVA04G01220	OMERI04G01560	KN540164.1_FGP003	BGIOSGA038246	OGLUM09G00370
CBSX6	CBSX6	LOC_Os01g44360	OBART01G23850	OB01G33220		ORUFI01G26680	OPUNC01G23900	ONIVA01G26810	OMERI01G21950	KN538828.1_FGP037	BGIOSGA004059	OGLUM01G27660
CBSCBS5	CBSX7	LOC_Os01g69090	OBART01G42010	OB01G50800	ORGLA01G0359900	ORUFI01G45370	OPUNC01G40900	ONIVA01G47160	OMERI01G39010	KN538700.1_FGP054	BGIOSGA000236	OGLUM01G46190
-	CBSX8	LOC_Os08g41740	OBART08G21410	OB08G28150	ORGLA08G0231800	ORUFI08G23880	OPUNC08G19500	ONIVA08G24390	OMERI02G01220		BGIOSGA026597	OGLUM08G22650
CBSX9	CBSX9	LOC_Os02g06410	OBART02G04490	OB02G13800	ORGLA02G0041600	ORUFI02G04640	OPUNC02G03750	ONIVA02G04500	OMERI02G05310		BGIOSGA007572	OGLUM02G04420
-	CBSX10	LOC_Os10g35630	OBART10G14660	OB10G21840		ORUFI10G15650	OPUNC10G13150	ONIVA10G16500	OMERI10G11390		BGIOSGA033216	
CBSX11	CBSX11	LOC_Os02g42640	OBART02G25440	OB02G32800		ORUFI02G26820	OPUNC02G23230	ONIVA02G27880	OMERI02G24920	KN539013.1_FGP003	BGIOSGA008689	OGLUM02G25950
CBSX12	CBSX12	LOC_Os04g58310	OBART04G29820	OB04G36790	ORGLA04G0261200	ORUFI04G31480	OPUNC04G27350	ONIVA04G28300	OMERI04G25210		BGIOSGA014082	OGLUM04G29730
-	CBSX13								OMERI02G33320			
-	CBSX14								OMERI01G33360			
-	CBSX15							ONIVA05G14030				
CBSX7/CBSCBS1	CBSCBS1	LOC_Os01g40420	OBART01G20960	OB01G30560		ORUFI01G23670	OPUNC01G21010	ONIVA01G23560	OMERI01G19150	KN538783.1_FGP013	BGIOSGA001328	OGLUM01G24630
CBSCBS2	CBSCBS2	LOC_Os01g69240	OBART01G42110	OB01G50980	ORGLA01G0361100	ORUFI01G45460	OPUNC01G41000	ONIVA01G47250	OMERI01G39130	KN538700.1_FGP089	BGIOSGA000232	OGLUM01G46320
CBSCBS3	CBSCBS3	LOC_Os04g31340	OBART04G10160	OB04G17440	ORGLA04G0072300	ORUFI04G11190	OPUNC04G08200	ONIVA04G07900	OMERI04G09350	KN539457.1_FGP003	BGIOSGA015211	OGLUM04G09770
CBSX10	CBSCBS4	LOC_Os01g44250	OBART01G23730	OB01G33160	ORGLA01G0192900	ORUFI01G26590	OPUNC01G23850	ONIVA01G26710	OMERI01G21900	KN538828.1_FGP039	BGIOSGA004055	OGLUM01G27560
-	CBSCBS5								OMERI05G12070			
-	CBSCBS6				ORGLA02G0341000							
-	CBSCBS7				ORGLA03G0390100							
CBSCLC1	CBSCLC1	LOC_Os01g65500	OBART01G39050	OB01G47840	ORGLA01G0329900	ORUFI01G42410	OPUNC01G37800	ONIVA01G43900	OMERI01G36000	KN539884.1_FGP008	BGIOSGA004909	OGLUM01G43320
CBSCLC2	CBSCLC2	LOC_Os01g50860	OBART01G27980	OB01G37140	ORGLA01G0231200	ORUFI01G31050	OPUNC01G27880	ONIVA01G31950		KN539741.1_FGP010	BGIOSGA004288	OGLUM01G31960
CBSCLC3	CBSCLC3	LOC_Os02g35190	OBART02G20670	OB02G28240	ORGLA02G0177300	ORUFI02G21700	OPUNC02G18580	ONIVA02G22650	OMERI02G20380	KN538737.1_FGP009	BGIOSGA006252	OGLUM02G20940
CBSCLC4	CBSCLC4	LOC_Os03g48940	OBART03G30570	OB03G37650	ORGLA03G0280100	ORUFI03G31810	OPUNC03G27870	ONIVA03G31920	OMERI03G26730	KN542832.1_FGP001	BGIOSGA009993	OGLUM03G30790
CBSCLC5	CBSCLC5	LOC_Os04g55210	OBART04G27250	OB04G34170	ORGLA04G0235000	ORUFI04G28900	OPUNC04G24730	ONIVA04G25550	OMERI04G22690	KN538912.1_FGP009	BGIOSGA017236	OGLUM04G27200
CBSCLC6	CBSCLC6	LOC_Os08g20570	OBART08G09810	OB08G18730	ORGLA08G0084800	ORUFI08G11100	OPUNC08G09140	ONIVA08G10550		KN538923.1_FGP002	BGIOSGA028422	OGLUM08G10690
CBSCLC7	CBSCLC7	LOC_Os12g25200	OBART12G10660	OB12G19240	ORGLA12G0099500	ORUFI12G11740	OPUNC12G09550	ONIVA08G11240	OMERI12G07260	KN540094.1_FGP001	BGIOSGA036265	OGLUM12G11730
CBSCLC8	CBSCLC8	LOC_Os08g38980	OBART08G19330	OB08G26380	ORGLA08G0170000	ORUFI08G21610	OPUNC08G17380	ONIVA08G21390	OMERI08G15970	KN539998.1_FGP007	BGIOSGA028930	OGLUM08G20420
CBSCLC9	CBSCLC9	LOC_Os02g48880	OBART02G30500	OB02G37640	ORGLA02G0260400	ORUFI02G32200	OPUNC02G28140	ONIVA02G33300	OMERI02G29500	KN539828.1_FGP002	BGIOSGA005723	OGLUM02G31200
CBSCLC10	CBSCLC10	LOC_Os04g36560	OBART04G13680	OB04G20940	ORGLA04G0107400	ORUFI04G14940	OPUNC04G11570	ONIVA04G11880	OMERI04G12300	KN538758.1__FGP045	BGIOSGA015026	OGLUM04g13430
-	CBSCLC11		OBART08G09800									
CBSSIS1	CBSSIS1	LOC_Os02g06360	OBART02G04440	OB02G13760	ORGLA02G0041100	ORUFI02G04580	OPUNC02G03710	ONIVA02G04440	OMERI02G05280	AMDW01038281.1_FGP001	BGIOSGA007570	OGLUM02G04360
CBSPPR1	CBSPPR1	LOC_Os09g26190	OBART09G11240	OB09G17770	ORGLA09G0083900	ORUFI09G12030	OPUNC09G09700	ONIVA09G10970	OMERI09G08760	KN538802.1_FGP030	BGIOSGA030810	OGLUM09G11610
CBSIMPDH1	CBSIMPDH1	LOC_Os03g56800	OBART03G36230	OB03G43120	ORGLA03G0332100	ORUFI03G37690	OPUNC03G33230	ONIVA10G12680	OMERI03G33460	KN538718.1_FGP015	BGIOSGA013663	OGLUM03G35940
CBSDUFCH1	CBSDUFCH1	LOC_Os03g39640	OBART03G24980	OB03G33260	ORGLA03G0231500	ORUFI03G25590	OPUNC03G22890	ONIVA05G20570	OMERI09G00570	KN539929.1_FGP005	BGIOSGA010327	OGLUM03G25680
-	CBSDUFCH2				ORGLA11G0223100							
CBSDUF1	CBSDUF1	LOC_Os05g32850	OBART05G15460	OB05G23380	ORGLA05G0133400	ORUFI05G16590	OPUNC05G13520	ONIVA05G15910		KN538789.1_FGP036	BGIOSGA019818	OGLUM05G16330
CBSDUF2	CBSDUF2	LOC_Os03g47120	OBART03G29240	OB03G36640	ORGLA03G0270900	ORUFI03G30380	OPUNC03G26710	ONIVA03G30450	OMERI03G25510	KN539376.1_FGP002	BGIOSGA013305	OGLUM03G29470
CBSDUF3	CBSDUF3	LOC_Os03g03430		OB03G11990	ORGLA03G0017900		OPUNC03G01830	ONIVA03G01670	OMERI03G01920	KN538922.1_FGP007	BGIOSGA011758	OGLUM03G02000
CBSCBSPB1	CBSCBSPB1	LOC_Os01g69900	OBART01G42210	OB01G51130	ORGLA01G0362200	ORUFI01G45630	OPUNC01G30700	ONIVA01G48290	OMERI01G39240	AMDW01119939.1_FGP001	BGIOSGA005080	OGLUM01G46480
CBSCBSPB2	CBSCBSPB2	LOC_Os11g06930	OBART11G04430	OB11G13640	ORGLA11G0041900	ORUFI11G04310	OPUNC11G04060	ONIVA11G04460	OMERI11G03910	KN538712.1_FGP055	BGIOSGA034423	OGLUM11G04170
CBSCBSPB3	CBSCBSPB3	LOC_Os01g73040	OBART01G44640	OB01G53940	ORGLA01G0384500	ORUFI01G48120	OPUNC01G43690	ONIVA01G50810	OMERI01G41500	KN541465.1_FGP002	BGIOSGA005225	OGLUM01G49030
CBSCBSPB4	CBSCBSPB4	LOC_Os12g07190	OBART12G04230	OB12G14180	ORGLA12G0039900	ORUFI12G04820	OPUNC12G04290	ONIVA12G03900	OMERI12G02390	KN538717.1_FGP087	BGIOSGA036554	OGLUM12G05000
CBSCBSPB5	CBSCBSPB5		OBART11G14240	OB11G21210		ORUFI11G15240	OPUNC11G11960	ONIVA11G13730	OMERI11G11890	KN538707.1_FGT010	BGIOSGA033955	OGLUM11G13800
CBSX8	CBSCBSCBD1	LOC_Os03g63940	OBART03G41570	OB03G48600	ORGLA03G0385000	ORUFI03G43350	OPUNC03G38580	ONIVA03G44080	OMERI03G38270	KN538745.1_FGP032	BGIOSGA013983	OGLUM03G41400
CBSCBS4	CBSCBSCBD2	LOC_Os04g32880	OBART04G11280		ORGLA04G0082300	ORUFI04G12420	OPUNC04G09270	ONIVA04G09180	OMERI04G09790	KN540832.1_FGP004	BGIOSGA015167	OGLUM04G10930
-	CBSTerCH										BGIOSGA039158	
-	CBSCoatomerE										BGIOSGA017237	

**Table 2:** The ratio of the number of non-synonymous substitutions per non-synonymous site (Ka) and the number of synonymous substitutions per synonymous site (Ks) in the same time period (Ka/Ks ratio) in duplicated gene pairs encoding CBS domain containing proteins in *Oryza* sp.

Genome	Paralogous Gene Pairs	Ka	Ks	Ka/Ks	Type of Selection	Type of Duplication
***O. barthii***						
OBART01G42110/OBART04G10160	ObCBSCBS2/ObCBSCBS3	0.318665	1.884767	0.169074	Negative	Segmental
OBART01G42010/OBART08G21410	ObCBSX7/ObCBSX8	0.418751	1.481156	0.282719	Negative	Segmental
OBART11G04430/OBART12G04230	CBSCBSPB2/CBSCBSPB4	0.13844	0.685464	0.201966	Negative	Segmental
OBART02G20670/OBART04G13680	CBSCLC3/CBSCLC10	0.073549	0.819377	0.089762	Negative	Segmental
OBART08G09810/OBART08G09800 *	ObaC-BSCLC6/ObaCBSCLC11	0	0			Tandem
***O. brachyantha***						
OB01G50980/OB04G17440	ObrCBSCBS2/ObrCBSCBS3	0.280887	2.127978	0.131997	Negative	Segmental
OB01G50800/OB08G28150	ObrCBSX7/ObrCBSX8	0.412242	1.49645	0.27548	Negative	Segmental
OB10G21840/OB02G32800	ObrCBSX10/ObrCBSX11	0.420248	0.90595	0.463875	Negative	Segmental
OB11G13640/OB12G14180	ObrCBSCBSPB2/ObrCBSCBSPB4	0.12394	0.770187	0.160921	Negative	Segmental
OB02G28240/OB04G20940	ObrCBSCLC3/ObrCBSCLC10	0.076766	0.80562	0.095288	Negative	Segmental
OB02G37640/OB08G26380	ObrCBSCLC9/ObrCBSCLC9	0.168039	1.389784	0.12091	Negative	Segmental
***O. glaberrima***						
ORGLA01G0361100/ORGLA04G0072300	OgCBSCBS2/OgCBSCBS3	0.312057	2.522365	0.123716	Negative	Segmental
ORGLA11G0041900/ORGLA12G0039900	OgCBSCBSPB2/OgCBSCBSPB4	0.148377	0.637685	0.232681	Negative	Segmental
ORGLA02G0177300/ORGLA04G0107400	OgCBSCLC3/OgCBSCLC10	0.073549	0.81441	0.09031	Negative	Segmental
***O. glumaepatula***						
OGLUM01G46320/OGLUM04G09770	OglCBSCBS2/OglCBSCBS3	0.301647	2.187936	0.137868	Negative	Segmental
OGLUM01G46190/OGLUM08G22650	OglCBSX7/OglCBSX8	0.512293	1.151735	0.444801	Negative	Segmental
OGLUM11G04170/OGLUM12G05000	OglCBSCBSPB2/OglCBSCBSPB4	0.152228	0.644932	0.236037	Negative	Segmental
OGLUM02G20940/OGLUM04G13430	OglCBSCLC3/OglCBSCLC10	0.085222	0.857666	0.099366	Negative	Segmental
***O. sativa* subsp. *indica***						
BGIOSGA000232/BGIOSGA015211	OsIbCBSCBS2/OsICBSCBS3	0.314794	2.122648	0.148302	Negative	Segmental
BGIOSGA000236/BGIOSGA026597	OsICBSX7/OsICBSX8	0.465974	1.220924	0.381657	Negative	Segmental
BGIOSGA033216/BGIOSGA008689	OsICBSCBS7/OsICBSX11	0.371022	0.695562	0.533414	Negative	Segmental
BGIOSGA034423/BGIOSGA036554	OsICBSCBSPB2/OsICBSCBSPB4	0.149293	0.639354	0.233506	Negative	Segmental
BGIOSGA006252/BGIOSGA015026	OsICBSCLC3/OsICBSCLC10	0.06991	0.819761	0.085281	Negative	Segmental
BGIOSGA008689/BGIOSGA014082	OsICBSX11/OsICBSX12	0.487428	0.914739	0.53286	Negative	Segmental
BGIOSGA017236/BGIOSGA017237	OsIC-BSCLC5/OsICBSCoatomerE	0.177	0.251	0.705	Negative	Tandem
***O. sativa* subsp. *japonica***						
LOC_Os01g69240/LOC_Os04g31340	OsJCBSCBS2/OsJCBSCBS3	0.310713	2.117603	0.146728	Negative	Segmental
LOC_Os01g69090/LOC_Os08g41740	OsJCBSX7/OsJCBSX8	0.481468	1.14693	0.419788	Negative	Segmental
LOC_Os11g06930/LOC_Os12g07190	OsJCBSCBSPB2/OsJCBSCBSPB4	0.152918	0.646455	0.236549	Negative	Segmental
***O. meridionalis***						
OMERI02G20380/OMERI04G12300	OmCBSCLC3/OmCBSCLC10	0.099376	0.823261	0.12071	Negative	Segmental
***O. nivara***						
ONIVA01G47160/ONIVA08G24390	OnCBX7/OnCBSX8	0.472698	1.145059	0.412816	Negative	Segmental
ONIVA11G04460/ONIVA12G03900	OnCBSCBSPB2/OnCBSCBSPB4	0.155831	0.642344	0.242597	Negative	Segmental
ONIVA02G22650/ONIVA04G11880	OnCBSCLC3/OnCBSCLC10	0.079511	0.825245	0.096349	Negative	Segmental
***O. punctata***						
OPUNC01G41000/OPUNC04G08200	OpCBSCBS2/OpCBSCBS3	0.303796	2.715368	0.11188	Negative	Segmental
OPUNC01G40900/OPUNC08G19500	OpCBSX7/OpCBSX8	0.440624	1.36049	0.323871	Negative	Segmental
OPUNC11G04060/OPUNC12G04290	OpCBSCBSPB2/OpCBSCBSPB4	0.165014	0.732182	0.225373	Negative	Segmental
OPUNC02G18580/OPUNC04G11570	OpCBSCLC3/OpCBSCLC10	0.061213	0.852646	0.071792	Negative	Segmental
***O. rufipogon***						
ORUFI01G45460/ORUFI04G11190	OrCBSCBS2/OrCBSCBS3	0.325525	2.010145	0.161941	Negative	Segmental
ORUFI01G45370/ORUFI08G23880	OrCBSX7/OrCBSX8	0.47554	1.099707	0.432424	Negative	Segmental
ORUFI10G15650/ORUFI02G26820	OrCBSX10/OrCBSX11	1.091475	2.108241	0.517719	Negative	Segmental
ORUFI11G04310/ORUFI12G04820	OrCBSCBSPB2/OrCBSCBSPB4	0.155928	0.646651	0.241132	Negative	Segmental
ORUFI02G21700/ORUFI04G14940	OrCBSCLC3/OrCBSCLC10	0.078198	0.750899	0.104139	Negative	Segmental

* The sequences of the pair are 100% identical.

## Data Availability

The datasets supporting the conclusions of this article are included within the article and its additional files. The sequence data for all the *Oryza* species were obtained from Gramene data resource (https://gramene.org (accessed on 15 September 2021)). For *O. sativa* subsp*. japonica*, the sequences were also retrieved from the RGAP (http://rice.plantbiology.msu.edu/ (accessed on 15 September 2021)).

## Supplementary Materials

The following supporting information can be downloaded at: https://www.mdpi.com/article/10.3390/ijms23031687/s1.
