# Supplementary material for: Genetic Conservation of CBS Domain Containing Protein Family in Oryza Species and Their Association with Abiotic Stress Responses
Source: Int J Mol Sci. 2022 Feb 1;23(3):1687. doi: 10.3390/ijms23031687 (PMC8836131; doi:10.3390/ijms23031687)
Supplement: Supplementary file 1 [file ijms-23-01687-s001.zip › ijms-1524801-supplementary.pdf]

International Journal of Molecular Sciences

MDPI

Supplementary materials

Genetic conservation of CBS domain containing protein family in *Oryza* species and their association with abiotic stress responses

Surabhi Tomar [1], Ashish Subba[1], Meenu Bala[2], Anil Kumar Singh[2,3], Ashwani Pareek[4,5] and Sneh Lata Singla-Pareek[1*]

[1]Plant Stress Biology, International Centre for Genetic Engineering and Biotechnology, New Delhi, 110067, India
[2]School of Genetic Engineering, ICAR-Indian Institute of Agricultural Biotechnology, Ranchi, 834010, India
[3]ICAR-National Institute for Plant Biotechnology, LBS Centre, Pusa Campus, New Delhi, 110012, India
[4]Stress Physiology and Molecular Biology Laboratory, School of Life Sciences, Jawaharlal Nehru University, New Delhi, 110067, India
[5]National Agri-Food Biotechnology Institute, Mohali, Punjab, 140306, India

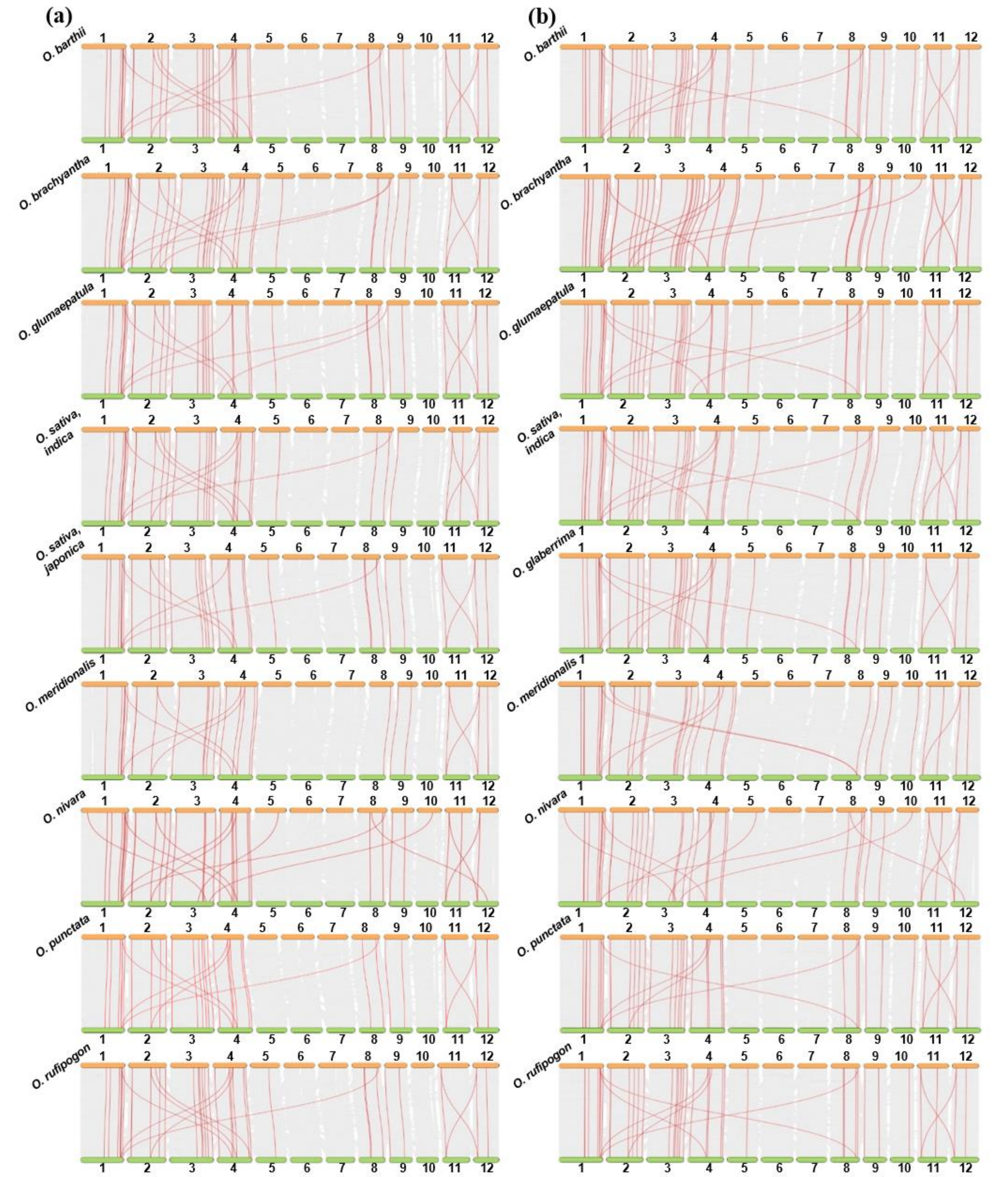

Supplementary Figure S1. Synteny of genes encoding CDCPs in different *Oryza* species in relation to the genes from two domesticated species, **(a)** *O. glaberrima* and **(b)** *O. sativa* subsp. *japonica*. The chromosomes of *O. glaberrima* and *O. sativa* subsp. *japonica* are shown in green colour in the respective panel, whereas the chromosomes from other species are shown in orange colour. Chr: chromosome.

Supplementary Table S1. Developmental and Stress-related *cis*-elements in the promoter regions of CDCPs

Gene Name	Motifs related to plant growth and development											Motifs related to stress response																				
	box4	G-box	circadian	SP1	CCGTCC-box	O_2-site	GT1	CAT-box	RY-element	GCN4_motif	ACE	CGTCA-motif	TGACG-motif	ABRE	E2Fb	Mybhv1	TCA-element	TGA-element	ARE	GARE-motif	P box	TC-rich repeats	MBS	LTR	GC Motif	ERE	MYB	WUN	AUXIN RR CORE	MYC	STRE	WRE3
	light responsive	light responsive	circadian control	light responsive	meristem specific activation	zein metabolism regulation	light responsive	meristem expression	seed-specific regulation	endosperm expression	light responsive	MeJA response	MeJA response	ABA response		Flavonoid Bisynthesis	heat stress/SA responsive	auxin-responsive	anaerobic induction	gibberellin-responsive	gibberellin-responsive	defense and stress	drought stress	low-temperature stress	Anoxia	Ethylene responsive		Wound	Auxin responsive		Stress responsive	Heat responsive
CBSX1	2	9	1	2		2	1			1	2	4	4	7	1	1	2	3	1	1			1	5		2	3	2	1	2	4	
CBSX2			1				1				1	5	5	7			2						1	2		2	7	1		6	3	
CBSX3	3	5		2			1					2	2	5			2		4								10			2	2	4
CBSX4	1	4				1	1	1				1	1	6		3	1	4					1			2					3	3
CBSX5	4		1		1				1		1	1	1				1		2				1	1		3	4	1		2	2	1
CBSX6	7			2		2		4		1		3	3	6				2	2		1		1				1		1	3	4	1
CBSCBS1	3	12		2			3				1	7	7	10				1	5	1					2	1	1			6	3	2
CBSCBSCBD1		4				2						1	1	4	1				2				1	1			6			2	1	2
CBSX9	2	5				2			1			3	3	5	2				1			1	1	1	1		4			4	2	1
CBSCBS4	2	7		4	2							2	2	7		1		1			1		3				7	1	1	1	1	4
CBSX11		4		2		1	2		1	1			5						2		2		9	1			14			4	1	1
CBSX12	6	16		1		2	2					4	4	7					1	2			2	2		1	6			3		1
CBSCLC1	2	3		1	1						5	5	5	2		1	2		1		1		3				10			1	4	2
CBSCLC2	1	6	2	4		1								6		1	2	1	2		3	1	1				5	1	1	3	3	1
CBSCLC3	4	2												3		1	1	1	1				3			2	4	1		6		2
CBSCLC4	5	6	1		1		2							4			2	1					1				5			1	4	1
CBSCLC5					1							1	1			1	1		1				2			2	7			5		3

CBSCLC6	3	3				1	2							2		1			4		1		2	1		1	1	1		3	6	1
CBSCLC7	2	4			1	3						1	1	5		1	2	1	2	1			4				8		1	7	4	
CBSCLC8	1	2			1							3	3	3			2	1	3	1			2			2	5	1	1	5	1	
CBSCLC9	2	3	1	1		2						3	3	3			3		2	1				6		2	1	2		2	3	1
CBSDUFCH1	1	2		2	1	2	1			1		2	2	4		1			1	1	1		3	6			9			2	5	8
CBSSIS1	1	7	1			1	4					2	2	5		1			2		1			1	1	2	2			4	1	1
CBSDUF1	2	8		2		2			2			2	2	10	1	1		1					2	1	2		3	2		5	4	1
CBSDUF2					1		1	1		1							2		2				5	2			5	1		7	3	1
CBSDUF3	3	9			1	1	3	1				3	3	8				1	3		1		2	2	3	1	5			2	3	2
CBSPPR1	1	2	1	1		1	2					4		2		1	2		1				1				8	1		6	4	
CBSIMPDH1	1	8		2	1			1				2	2	6		1			4		1		1	1		3	3			4	3	2
CBSCBS2	3	3				1						1	1	3					2						1		2			5	2	3
CBSCBS3	1	3		1	1			1				7	7	3					2		1		1							3	2	3
CBSCB-SCBD2	2	6	1			1						5	5	6				2	4	2			2			1	5	1		1	4	2
CBSX7	5	2				2						4	4	2					1	1						3	3			6	3	2
CBSX8	3	4	1				2					6	6	3		1		1							1		2	1	1	3		1
CBSX10		2										3	3	3					1	1			1	1	1	1	2			1		2
CBSCBSPB1	1	11		6	1	1		1					12			1	2		1				1				3				7	2
CBSCBSPB2	1	8			1	2		1			5	5		7					1			1	1	1		1	7			2	4	1
CBSCBSPB3	5	6			2	1	1	2				1	1	6		1			1		2		1	1		1	5			5	1	1
CBSCBSPB4		7					1					1		7			2		4		2						2			4	1	1
